# Robust and High-Resolution All-Ion Fragmentation LC-ESI-IM-MS Analysis for In-Depth Characterization or Profiling of Up to 200 Human Milk Oligosaccharides

**DOI:** 10.1021/acs.analchem.4c06081

**Published:** 2025-03-06

**Authors:** John Gonsalves, Julia Bauzá-Martinez, Bernd Stahl, Kelly A. Dingess, Marko Mank

**Affiliations:** 1 Danone Research & Innovation, Uppsalalaan 12, 3584 CT Utrecht, The Netherlands; 2 Skid Visual Science, Castaños 106 1B, Mataro, 08302 Barcelona, Spain; 3 Utrecht Institute for Pharmaceutical Sciences, Department of Chemical Biology & Drug Discovery, Utrecht University, 3584 CG Utrecht, The Netherlands

## Abstract

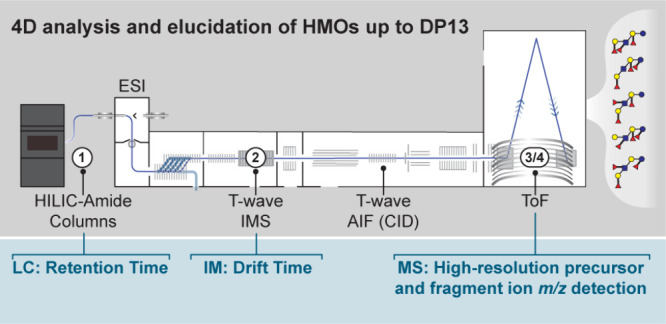

Human milk oligosaccharides (HMOs) represent the third most abundant fraction of biomolecules in human milk (HM) and play a crucial role in infant health and development. The unique contributions of HMOs to healthy development of breast-fed infants are assumed to rely on the extraordinary complexity and diversity of HMO isomeric structures, which in turn still cause a huge analytical challenge. Many contemporary analytical methods aiming for more detailed HMO characterization combine ion mobility (IM) with LC-MS for enhanced structural resolution but are typically lacking the robustness necessary for application to HM cohorts with hundreds of samples. To overcome these challenges, we introduce a novel, robust all-ion fragmentation (AIF) LC-ESI-IM-MS method integrating four analytical dimensions: high-resolution LC separation, IM drift time, accurate mass precursor, and fragment ion measurements. This four-dimensional (4D) analytical characterization is sufficient for resolving various HMO structural isomers in an efficient way. Thereby, up to 200 HMO compounds with a maximum degree of polymerization of 13 could be simultaneously identified and relatively quantified. We devised two methods using this 4D analytical approach. One intended for in-depth characterization of multiple known but also novel HMO structures and the second is designed for robust, increased-throughput analyses. With the first approach, five trifucosyl-lacto-*N*-tetraose isomers (TF-LNTs), four of which were never detected before in HM, as well as additional difucosyl-lacto-*N*-heaose isomers (DF-LNHs), were revealed and structures fully elucidated by AIF and IM. This exemplifies the potential of our method for in-depth characterization of novel complex HMO structures. Furthermore, the increased-throughput method featuring a shorter LC gradient was applied to real-world HM samples. Here, we could differentiate the HM types I–IV based on a broader range of partly new marker HMOs. We could also derive valuable new insights into variations of multiple and rare HMOs up to DP 11 across lactational stages. Overall, our AIF LC-ESI-IM-MS approach facilitates in-depth monitoring and confident identification of a broad array of distinct and simple to very complex HMOs. We envision this robust AIF LC-ESI-IM-MS approach to advance HMO research by facilitating the characterization of a broad range of HMOs in high numbers of HM samples. This may help to further extend our understanding about HMOs structure–function relationships relevant for infants’ healthy development

## Introduction

HM is the gold standard choice for infant nutrition^1^ since it contains all of the components required for optimal infant nutrition, health, and development.^2,3^ The World Health Organization and pediatric societies recommend breastfeeding exclusively for at least the first 6 months of life, which benefits both the infant and the mother.^4^ Moreover, HM is dynamic and tailor-made, as its composition is thought to adapt to the nutritional, immunological, and developmental needs of the infant throughout lactation.^5,6^

After lipids and lactose, HMOs constitute the third largest biomolecular fraction of breast milk^7^ and they may carry out many fundamental functions for infant health and development. HMOs are largely indigestible,^8^ and thus, they reach the large intestine, where they carry on three main functions: they promote the development of a healthy microbiome through their probiotic role;^9−11^ modulate the neonatal immune system by, e.g., influencing the expression of inflammatory markers;^12^ and contribute to fight infection, as they prevent the adherence of pathogenic bacteria and viruses by modifying the glycocalyx of intestinal epithelial cells^13^ or by acting as decoys.^14,15^ Additionally, HMOs are also thought to contribute to the infant’s cognitive development.^16,17^

HMOs are a mixture of structurally divergent, unconjugated water-soluble oligosaccharides produced and secreted by the mother’s mammary gland during lactation.^18,19^ HMOs are built to a varying degree of polymerization (DP) from five monosaccharides: d-glucose (Glu), D-galactose (Gal), L-fucose (Fuc), *N*-acetylglucosamine (GlcNAc), and the sialic acid derivative *N*-acetyl-neuraminic acid (Neu5Ac).^20,21^ As a complex and structurally diverse group of molecules, every mother produces a distinct set of HMOs, which are strongly, but not exclusively, influenced by the maternal genotype on the Lewis (Le) and Secretor (Se) genes (Le/Se).^22,23^ Se (FUT2) and Le (FUT3) genes encode the enzymes α1–2-fucosyltransferase (Se) and α1–3/4-fucosyltransferase (Fuc-TIII), which shape the biosynthesis of fucosylated HMOs and, therefore, play a fundamental role in the generation of the final HMO repertoire.^24^ Moreover, within one mother, HMO concentrations also have been reported to vary throughout lactation and even throughout the day.^25^ Interestingly, a recent publication^26^ showed that the HMO profile from the same women across repeated pregnancies was highly conserved across lactation.

To date, around 162 HMO structures have been characterized^27,28^ using various analytical techniques, including (liquid) chromatography (LC), mass spectrometry (MS), or nuclear magnetic resonance (NMR), and quantified with analytical techniques such as capillary electrophoresis coupled to laser-induced fluorescence (CE-LIF) or high-performance anion exchange chromatography/pulsed amperometric detection (HPAEC PAD).^29−41^ Nevertheless, early MS-based investigations^30,42^ already anticipated over 1000 structural variants, including low- and high-molecular-weight species ranging from DP 3 to up to DP > 40. In fact, the structural and compositional characterization of HMOs has long been challenging, largely due to the structural diversity, but also to their broad dynamic range. With the top 10 most abundant HMOs constituting 70% of the total concentration in HM,^43^ detection of less abundant but maybe still biologically relevant HMO species challenges instrument sensitivity and selectivity. Moreover, the inherent structural complexity of HMOs, characterized by subtle differences in monosaccharide composition, (anomeric) linkages, and branching patterns, leads to co-occurrence of multiple structural and steric isomers. In fact, it is in these subtle structural and quantitative differences that the functionality of HMOs may lie. For example, structural differences in HMO species could imply differing biological activities by prompting distinct HMO-receptor interactions.^44^ Therefore, understanding qualitative and quantitative HMO complexity, both inter- and intraindividual, is vital for tailoring infant formulas to better replicate the content, and thus the nutritional and developmental properties, of HM.

In recent years, advancements in glycomic technologies, specifically on high-resolution UHPLC and LC-MS/MS methodologies,^45,46^ have enabled more comprehensive analyses of individual HMO structures. Due to its high sensitivity, specificity, and quantitative potential, LC-MS/MS is the gold standard for HMO characterization.^46−48^ However, the vast isomeric complexity of HMOs often constrains LC-MS/MS, leading to insufficient resolution at LC or MS/MS levels and hindering the detection and quantification of all isomeric species.^49,50^ Prioritizing throughput is essential for large-scale studies and routine analyses of HMO research. Recent publications have made impressive advancements in the identification of significant numbers of HMOs (up to 154 in some cases), but they generally rely on either derivatization, lactose removal, and/or fractionation, hindering throughput.^38,51−59^ Moreover, these approaches focus on identification based on spectral evidence and not on monitoring the concentration trajectories of the individual HMOs over the stages of lactation in different donors.

One potential solution to the bottlenecks of throughput and isomeric complexity could come with IM spectrometry, to add analysis dimensionality without increasing the run time. IM can efficiently separate coeluting isomeric ions based on their electrophoretic mobility in a gas matrix. By reflecting differences in collisional cross-section (CCS) or drift times, IM can improve ion separation and characterization. Some groups have developed workflows centered around IM for oligosaccharide analyses in HM^60−64^ although, to the best of our knowledge, just one study has reported a synergistic, hyphenated use of LC, IM, and MS for oligosaccharide analysis.^65^ However, all of these studies have only been applied to monitor a few HMOs, missing the broader picture of HMO diversity in HM.

Here, we developed an integral AIF LC-ESI-IM-MS workflow optimized for HM samples, capable of providing a broad perspective of the HMO repertoire. By combining high-resolution retention time (RT) information along with IM drift time information and accurate precursor and fragment ion mass measurements, our hyphenated AIF LC-ESI-IM-MS workflow can simultaneously identify and quantify changes in a vast array of HMOs, spanning 2 to 13 DP. We propose two modalities of our AIF LC-ESI-IM-MS workflow: an in-depth screening mode with a longer method (and a total of 108.5 min per sample), capable of detecting up to 203 HMOs in DP range 2 to 13, and a robust increased-throughput mode with a shorter method (and a total of 59.5 min per sample), which can monitor up to 133 HMOs ranging from 2 to 11 in DP. Importantly, the increased-throughput mode was conceived to swiftly monitor the HMO abundance and composition of HM in bigger cohorts, aiming to detect and relatively quantify biologically or functionally relevant features. Altogether, these methods will help improve our understanding of the HMO composition in HM and help accelerate the development of tailored milk preparations.

## Experimental Methods

### HM Samples and Donors

To evaluate the method, HM standard reference material 1953^55^ was used (NIST, National Institute of Standards and Technology). For milk-type characterization, HM samples were collected from four healthy donors at weeks 16–20 postpartum. Longitudinal HM samples were collected from one healthy donor at days 2, 3, 10, 22, 47, and 201 postpartum. From all samples, 100 μL aliquots were separated and stored in 500 μL microtubes (Thermo Scientific, 3743) at −20 °C until further processing. Written informed consent was obtained from donors prior to sample collection. All samples used were donated to Danone Research & Innovation in accordance with the Helsinki Declaration II.

### Sample Preparation and Cleanup

Aliquots from milk samples were thawed for 30 min (min) at room temperature before addition of 300 μL of 1.33% formic acid (FA) (Biosolve, 069141A8) and 0.098 mg/mL arabinopentaose (Megazym, O-APE) as internal standard, which was incorporated by 30 s (s) vortexing. To separate the lipidic fraction, samples were incubated for 60 min at 4–8 °C, after which samples were spun down at 1500*g* for 15 min at 4 °C. A hole was carefully pierced on the lipid layer using a 200 μL 8-channel pipet and, using a clean pipette tip, 75 μL of delipidated milk was transferred to a 96-well LoBind 2 mL plate (Eppendorf, 0030 504.305) and diluted with 675 μL of Milli-Q Water (Millipore, IQ7000).

For further sample cleanup, Oasis mixed cationic exchange (MCX) solid-phase exchange (SPE) 96-well plates (Waters,186000250) were used, and extraction was vacuum assisted on a Manifold (Merck, 575650-U). First, MCX SPE microcolumns were conditioned by sequential addition of 1 mL of 2% ammonium hydroxide (NH_4_OH) (Merck, 5.33003.0050) in 60% acetonitrile (ACN) (VWR, 83640.290), 1 mL of 1% FA, in 60% ACN, 1 mL of 0.1% FA in water, and finally 1 mL of ACN. After conditioning, microcolumns were dried by applying vacuum for 5 min. Then, 450 μL of the diluted samples were loaded on the microcolumns, and the flowthroughs, containing the clean HMO fraction, were collected and 150 μL was diluted with 300 μL of ACN. These extracts were then vortexed for 30 s at 1500 rpm, and residual proteins precipitating upon ACN addition were removed by a final centrifugation at 1500*g* for 15 min at 21 °C. Afterward, 150 μL of clean HMO extracts were transferred to a MaxPeak 300 μL 96-well plate (Waters, 186009186) and sealed using a polyester heat seal (Waters, 186002788). Note that the final dilution factor of HM was ∼1:120.

### Generation of an Extensive Calibration Reference from Isolated HMO Fractions and Commercially Available HMO Standards

Of the hundreds of identified HMOs, only a few short-chain or low-molecular-weight HMOs are commercially available, and even then, their cost and purity can be a bottleneck.^48^ To circumvent this, we generated an HM-isolated total HMO fraction to serve as a global calibration reference, with virtually all possible HMO structures contained. Here, pooled HM was used to isolate two HMO fractions, one with neutral and one with neutral and acidic HMOs as described elsewhere.^30^ The two fractions were combined and used to perform relative quantitation of the HMOs in HM. These isolated fractions were used to optimize the chromatographic separation and structure elucidation. The identity of the HMOs in the isolated fractions was annotated for HMOs for which pure standards were commercially obtained. Moreover, these commercial standards are instrumental in determining the expected elution time, drift time, exact mass, and fragmentation spectrum of individual compounds present in the calibration reference and HM samples. To accurately determine these parameters, all commercial standards were prepared at a concentration of 0.01 mg/mL and injected individually using the parameters and gradients described in the next section. Notably, if more standards with higher purity become commercially available in the future, then these can be individually injected, and their concentration can then be retrospectively calculated within the calibration reference without the need for reanalysis. The 46 commercial HMO standards used, their providers, and their catalog number and structure are summarized in Table S1.

### AIF, Liquid Chromatography Coupled to Negative-Mode Electrospray Ionization, IM Separation, Mass Spectrometry (AIF LC-ESI-IM-MS) for HMO Analysis

The instrumental analysis of HMOs was performed on a Vion-IM Qtof MS instrument (Waters, instrument driver 3.1) coupled to an Acquity UPLC I class plus system (Waters). The 96-well plate containing the clean HMO extracts was placed into the autosampler and kept at a constant temperature of 15 °C. For all injections, 6.5 μL of the sample was loaded into the sample loop with a syringe draw rate of 30 μL/min, cushioned by 2 μL of air, and injected into the two interconnected Acquity UPLC Premier Glycan Amide columns (2.1 mm × 150 mm internal diameter, 1.7 μm particle size, and 130 Å pore size from here-on HILIC-Amide, Waters, 186009976 and 186009524). Optimal operational conditions were determined and are described in detail in the Supporting Information, section 1. Briefly, HMO separation was achieved at a constant temperature of 67.5 °C. The two in-series HILIC-Amide columns were operated using the two methods devised for either in-depth characterization of HMOs (from here-on long method, 108.5 min per sample, including system lockmass check, injection, and run time) or increased-throughput analyses (from here-on short method, 59.5 min per sample, including system lockmass check, injection, and run time). The gradients and buffers used for the long and short methods are thoroughly detailed in Supporting Information section 1b.

After separation, HMOs were ionized by electrospray ionization (ESI) prior to being placed in the mass spectrometer. Relevant parameters where optimization was necessary are detailed in Supporting Information section 2. Briefly, after thorough optimization, ESI was performed on a LockSpray source operated in the negative ion mode under the following optimal parameters: source temperature, 120 °C; capillary voltage, 0.5 kV; source offset voltage, 80 V; cone voltage, 40 V and cone gas flow, 50 L/h; and desolvation temperature, 550 °C and desolvation gas flow, 1000 L/h. The soft transmission mode was activated together with the following advanced settings: StepWave offset was set to 20 V for step 1 and to 30 V for step 2, while velocity and pulse height were kept at 300 ms and 15 V, respectively, for both steps; StepWave RF and Trap/IM RF were kept at 250 V; ion gain RF offset was set to 200 V at a gain of 5; the first cell RF was set to 300 V, and the second cell RF was set to 175 V and a gain of 1; finally, to avoid ions of *m*/*z* < 400 entering the collision cell, the MS profile mode was set to “Fixed”, with the quadrupole set to 500 *m*/*z*.

The TOF analyzer was operated in the sensitivity mode. For each cycle, four independent MS scans were sequentially acquired within the mass range 55–2000 *m*/*z*. First, ions were accumulated for 400 ms, and intact mass spectra were recorded in the TOF, using 6 eV to improve transmission through the collision cell. After intact mass analysis, fragmentation was achieved by collision-induced dissociation (CID). Two strategies of fragmentation were used for the short and long methods. The long method was operated with a ramped collision energy of 30–70 eV for improved data processing purposes. In this manner, retention and drift time deconvoluted spectra can be obtained for spectral elucidation. For the short method, which is operated as targeted method for monitoring individual HMOs, mass spectra from three individual fragmentation events were recorded, at 30 eV for 500 ms, 50 eV for 500 ms, and 75 eV for 300 ms. Depending on the desired fragment to be monitored, a single fragmentation event can be manually selected during data processing. An individual collision event resulted in more sensitivity over the ramped collision energy approach.

Note that lockmass correction was performed every 10 min at a flow of 10 μl/min, using a leucine enkephalin (LeuEnk) solution. Mass calibration and CCS calibration of the system were done using Major Mix (Waters, 186008113). The Vion-IM resolving power is approximately 20.

### Processing of Data

**Figure 1:**
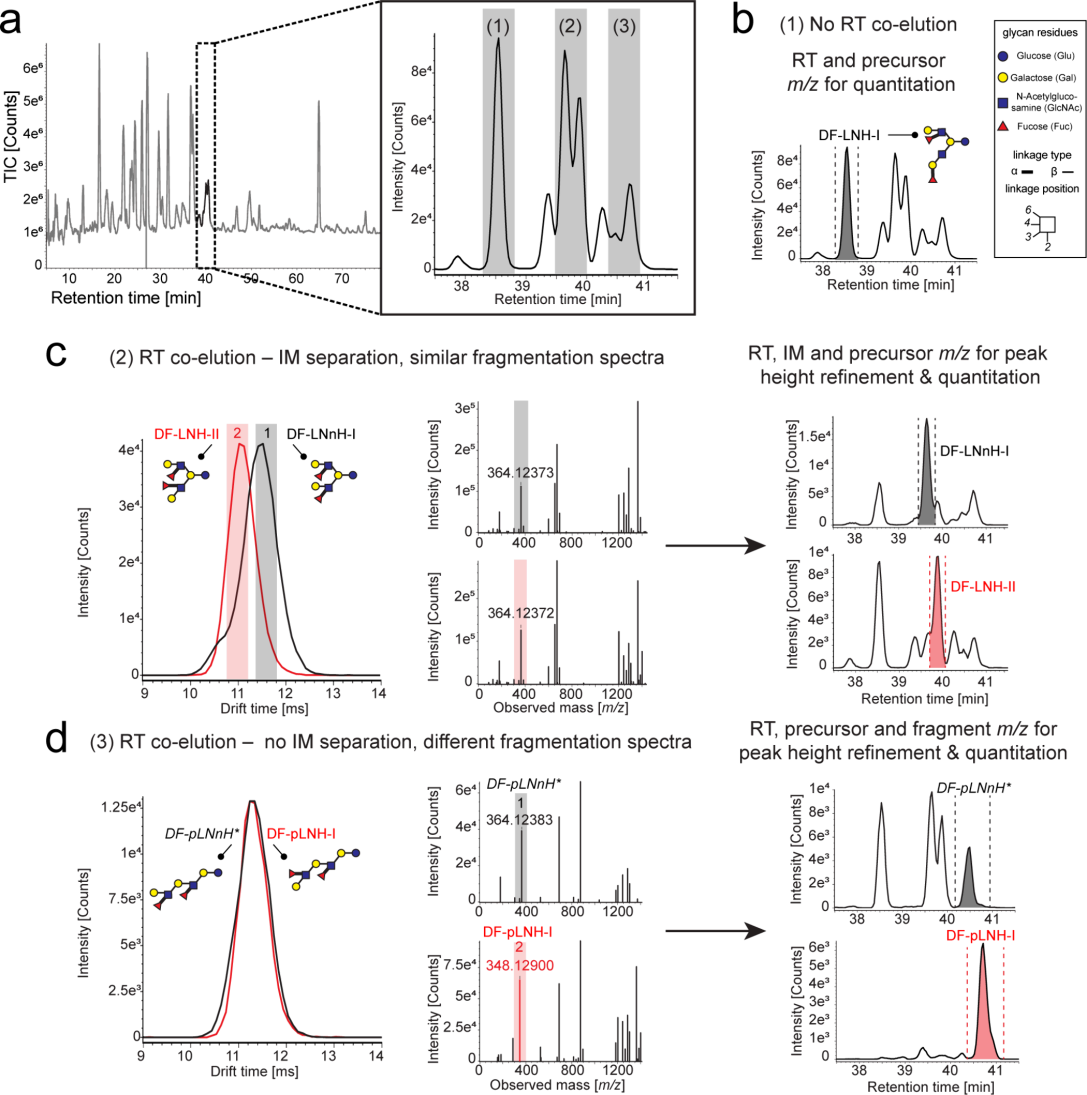
AIF LC-ESI-IM-MS processing workflow for in-depth HMO characterization. (a) The total ion current chromatogram (TIC) is displayed, with a zoom-in of the XIC of the [M+COOH]– adduct of the DF-LNH isomer series. Three peaks are highlighted to demonstrate the separation power across the four dimensions of this AIF LC-ESI-IM-MS method. (b) Example of retention time (RT)-based peak selection, which is possible when no isomers coelute. In this case, RT and high-resolution accurate mass detection of the precursor ion are used for identification and quantitation. (c) Example of drift-time-based peak refinement for the isomeric HMO species DF-LNH-I (gray shading) and DF-LNnH-II (red shading). Although these species coelute and display near-identical fragmentation spectra, the two isomers can be separated based on drift time on the IM dimension, enabling peak height refinement and quantitation through RT, IM, and precursor ion *m*/*z* detection. (d) Example of accurate mass detection-based peak refinement for the isomeric HMO species DF-LNH-X3 (gray shading, named DF-pLNnH after spectral elucidation displayed on Figure S10) and DF-pLNH-I (red shading). Although these species coelute and display near-identical drift time on the IM dimension, the two isomers can be separated based on compound-specific diagnostic fragments (*m*/*z* = 364.128 and *m*/*z* = 348.129, respectively, for DF-LNH-X3 (DF-pLNnH) and for DF-pLNH-I), enabling peak height refinement and quantitation through RT, IM, and fragment ion *m*/*z* detection. Monosaccharide symbols and structural representations of HMOs were drawn in Illustrator according to the symbol nomenclature for glycans (SNFG). Linkage type is represented by line thickness (α, thick line and β, thin line), while linkage position is represented by the orientation of the line.

Processing of mass spectral raw data was performed using the UNIFi software (v_1.9.4, Waters). For analyses done using the long method, the HDMSe Workflow was used for RT and drift time deconvolution, including the following settings: 4D peak detection was set to automatic; the low and high energy thresholds were set to 500 and 200 counts, respectively; and background filtering was set to “high”. For 4D isotope clustering, the fraction of the chromatographic peak width for cluster creation and for high-to-low energy association was set to 0.2. The fraction of the drift peak width was set to 0.15 for cluster creation and 0.3 for high-to-low energy association. High-to-low energy association was set at 200 counts. A maximum charge of 4 and up to 6 isotopes was set to define a cluster. Target by mass was activated, with a target match tolerance of 10 ppm and a fragment match tolerance of 10 mDa. Also, 5% tolerance at the CCS level was used to select targets. The following adducts were selected: [M-H]–, [M+HCOO]–, [M-2H]2–, [M+2(HCOO)]2–, [M-Glc+HCOO]–, and [M-Glc-Fuc+HCOO]–. For analyses performed using the short method, the 2D ToF Workflow was used. Extracted ion chromatograms (XICs) were generated with a 50 ppm extraction window of the adduct/fragment and a 0.3 ms drift time window. Retention time window was set to ±0.1 min. To determine the changes in HMO composition over the course of lactation (see Figure 4A–C), peak height was extracted for the most abundant adduct. For monitoring compound stability over time (Figure S1E–G) on either HILIC-amide or PGC stationary phases, a peak height or area was used. Specifically, for isomers that coelute, peak area is hard to determine. In these cases of interference, after refining the peaks based on the 4D approach as detailed in Figure 1, peak height was preferentially utilized instead of peak area since it provides a more reliable estimate of compound abundance. Finally, the limit of detection (LOD) of this method is estimated to be of at least 1–5 mg/L, based on the method’s ability to detect β3′-galactosyllactose in SRM1953 milk. The concentration of this glycan in mature HM is reported to be of 1–5 mg/L in literature.^66^

Data formatting and visualization were done using Excel and R Studio (version 2022.10), using R (version 4.2.2). The R libraries used were ggplot2, tidyverse, dplyr, scales, gridExtra, readxl, ggrepel data.table, and ggbreak. Figures were edited by using Adobe Illustrator (version 28.0).

### Data Availability

Raw data supporting the findings presented in this paper can be made available upon reasonable request to the corresponding author. Data from which figures were generated is available in Tables S1 and S2, as well as from the FigShare repository (DOI: 10.6084/m9.figshare.27269076).

## Results and Discussion

### 4-Dimensional AIF LC-ESI-IM-MS Analysis Enables Clean Isomer Discrimination for In-Depth and Accurate HMO Analysis

The broad diversity of HMOs found in HM, although key for infant nutrition and development, represents an important analytical challenge. To obtain a complete picture, sufficient separation of the HMOs is needed to maximize resolution for further characterization, but the co-occurrence of many structural isomers and a large concentration range challenge this separation. Moreover, in oligosaccharides, the presence of anomeric carbon leads to the formation of two different anomers, which can result in two separate chromatographic peaks for the same compound. This phenomenon complicates quantification and identification. Therefore, conditions that promote the collapse of the anomer peaks into a single peak are preferred.

While derivatization of oligosaccharides prior to chromatography has been used to avoid splitting of HMO-anomers into two peaks,^67^ this comes at the cost of additional sample processing steps and time. Thus, with throughput and versatility in mind, we opted for a derivatization-free approach to achieving anomer collapse. Alternatively, utilizing different methodological conditions such as the stationary phase of choice, specific pH, and temperature can promote anomer peak collapse. Here, we worked with two commonly used stationary phases in oligosaccharide analyses: PGC and HILIC-amide columns.^48^ Although HILIC-amide can provide robust glycan separations,^68^ PGC columns are known to provide better isomer separation,^69^ especially for HMOs with high DP. To achieve anomer collapse while maximizing compound stability over time, we optimized the operational conditions and compared HILIC-amide and PGC columns for HMO separation, using both an extensive calibration reference standard and the SRM1953 HM.^55^ The results of these optimizations are thoroughly described in the Supporting Information.

Briefly, HILIC-amide was selected as the optimal stationary phase for robust HMO analysis due to the DP-dependent compound elution profile (Figure S1A,B), the similar isomer separation power (Figure S1C,D), and the greater stability (Figure S1F,G) when compared to PGC columns. Notably, the coefficient of variation (CV) was very low for most eluted HMOs, on HILIC columns, with 65% of structures below 10% CV (Figure S1E). HILIC-amide was beneficial for the separation of acidic HMOs, but our data show that the number of detectable HMO isomers varied between both stationary phases (Table S2). This is attributed to differences in HMO physicochemical properties, which have an influence in their elution patterns. Finally, 197 and 203 HMOs in the DP range 2–13 could be detected in the global HMO standard derived from pooled HM with either PGC or HILIC-amide columns, respectively (Table S2). To the best of our knowledge, this is the deepest analytical coverage of HMO structural diversity in HM by an LC-MS approach regarding both the number of detected HMO structures and the maximum degree of HMO-polymerization (DP), i.e., HMO size. Thus, our method might be considered as relevant for the improvement of HMO or free glycan analysis, pushing performance limits beyond achievements recently reported.^38,51−59^

A frequent problem in HMO analysis by LC-MS is sample complexity and concentration range, which requires prefractionation. However, prefractionation methods are usually not compatible with higher throughput studies, where a single injection is preferred to minimize the run time. To circumvent the need of fractionation, another dimension of analysis can be added by using IM. With our AIF LC-ESI-IM-MS setup (Figure S2A, gray), the four dimensions (4D) of the analysis (i.e., LC, IM and AIF, including accurate precursor and fragment ions mass) allow for case-tailored isomer selection and peak refinement, enabling accurate identification and quantitation. With our processing workflow, HMOs are discerned based on at least one of the four levels of separation: LC-derived RT, IM-derived drift time, or AIF-derived exact mass measurements of precursor and diagnostic ion fragments. Note that although the AIF dimension provides information at both precursor and fragment ion levels, the precursor ion masses can be used to corroborate the presence of isomers through exact mass measurements, while the fragment-ion masses are the ones specific to each isomer and thus allow differentiation and separation of isomeric species. Importantly, the third (precursor ion) and fourth (fragment ion) dimensions are interlaced and cooperate to provide accurate separation and identification of isomeric HMO species. This approach can simultaneously monitor up to 200 HMOs from HM, removing the need for prefractionation. The 4D selection scheme is illustrated in Figure 1 for the DF-LNH isomer series. Using the robust short method (59.5 min per sample), we detect seven isomers of DF-LNHs based on exact precursor ion mass that elute from minute 39 to 41 (Figure 1A and Table S2), but this relatively narrow elution interval makes it complicated to isolate some of the isomers to achieve accurate identification and quantitation. While some isomers like DF-LNH-I do not display RT coelution and can thus be directly quantified using RT and precursor *m*/*z* (Figure 1B), other isomers coelute (Figure 1C,D) and other dimensions are required for peak height refinement and quantitation. For instance, for difucosyl-lacto-*N*-*neo-*hexaose I (DF-LNnH-I) and difucosyl-lacto-*N*-hexaose II (DF-LNH-II) (Figure 1C), separation is suboptimal at RT and fragment ion level, since these isomers partly coelute leading to peak overlap and result in similar diagnostic fragments ions upon CID fragmentation. However, DF-LNnH-I and DF-LNH-II do separate based on drift time, allowing for distinction and peak refinement, which enables identification and quantitation of either species. Similarly, a measured DF-LNH isomer, tentatively named DF-LNH-X3, markedly coelutes with the well-known difucosyl-*para-*lacto-*N*-hexaose I (DF-pLNH-I) (Figure 1D). In this case, these two isomers are suboptimally separated by either RT or drift time. However, their fragmentation spectra do differ significantly, reflecting differences in positioning of the fucoses within the HMO backbone. Indeed, two differential diagnostic fragment ions (*m*/*z* = 364 and *m*/*z* = 348)^40,44,70,71^ are formed, and these enable distinction, peak refinement, and quantitation. To this, we can add up the previously described formate adduct generation, which enables distinction between coeluting neutral and acidic HMOs (Figure S3A–D). Overall, with this approach, a plethora of HMOs can be confidently distinguished in HM samples, enabling cross-comparison of present species and their relative quantities. It must be noted that the overall response is, approximately, a factor of 5 lower when selecting IM CID fragments compared to IM intact ions due to the fragmentation losses.

### High-Accuracy AIF CID Fragmentation MS Analysis for Structural Elucidation of Known and Novel HMOs

One of the key attributes of a method intended to understand the composition of HMOs is its ability to provide not only information on the number of HMO species and their relative quantity present in a given sample but also to deliver sufficient evidence to understand the structural composition of known and new isomers observed. CID fragmentation combined with high-accuracy mass analysis at precursor and fragment ion levels has been proven instrumental in this process.^40,44,70−72^ In our method, we implemented an integrative fragmentation strategy capable of rendering informative fragments from HMOs regardless of their size and charge. First, a scan is acquired at a very low 6 eV collision energy (Figure S2B), which delivers accurate intact mass measurements of deprotonated ions (Figure S3A–E, green). In the case of neutral HMOs, deprotonated ions frequently co-occur with more abundant formate adducts (Figure S3A,B, gray). Next, either a collision energy ramp optimized for the range of accessible HMOs (long method) or three consecutive MS scans (short method) are acquired at three selected collision energies. Thereby, informative fragmentation spectra are generated. At 30 eV, fragmentation is achieved for lower DP *neutral* HMOs. At 50 eV, fragmentation is achieved for higher DP *neutral* HMOs. Finally, at 75 eV, sufficient fragmentation of acidic HMOs takes place, leading to the formation of an informative sialic acid fragment at *m*/*z* = 290.^73^ An overview of all the settings optimized to achieve in-depth HMOs characterization in HM on an AIF LC-ESI-IM-MS system is displayed in Figure S2B. Note that although AIF is known to generate more complex spectra than data-dependent acquisition (DDA) or targeted approaches, RT and IM can be used to achieve spectral deconvolution and significant reduction of final AIF data complexity. While AIF could sacrifice some sensitivity compared to DDA or targeted methods, it offers the advantage of allowing post hoc data reinterrogation without additional sample preparation or reinjection. Moreover, and despite their higher selectivity and sensitivity, DDA and targeted can miss coeluting low abundance or isobaric compounds due to limitations like insufficient precursor coverage by usage of dynamic exclusion. Overall, and despite its limitations, which are counterbalanced by the multidimensionality of our approach, AIF represents a more versatile option for comprehensive HMO analysis.

The information contained within the fragmentation spectra, mostly in the form of glycosidic (B/Y and C/Z) and cross-ring (A/X) fragment ions,^74^ is, in most cases, comprehensive enough to provide sufficient evidence as to distinguish the structure and linkages of known and novel HMOs. In more detail, especially the presence of A-, D-, and C-fragments in the AIF CID spectra, as exemplified in Figures S4–S15, allows for a clear first structural elucidation of the respective TF-LNT isomers. The same types of fragments, which have been described to be crucial for structural elucidation of complex glycans in previous publications (see *e.g.,* Mank et al. 2019,^40^ Mank et al. 2020,^44^ Pfenninger et al. 2002,^70^ Chai et al. 2001,^75^ Spengler et al. 1990,^76^ and Hofmeister et al. 1991^77^), are also of key importance to elucidate structures of other complex HMOs like the different DF-LNH isomers or LSTa (see Figures S4–S10 and S15). Furthermore, the validity of (novel) HMO structures derived from AIF CID was also further confirmed based on known rules for glucosyltransferase activity in HMO catabolism.^24,78^ In the case of DF-LNDHs (see Figures S4–S10), the specific fragment ions obtained facilitate distinction of the coeluting isomers DF-pLNH-I (Figure S8) and DF-LNH-X3 (Figure S10), previously described in Figure 1D. This eventually allows us to identify DF-LNH-X3 as DF-pLNnH,^55^ and to assign the right structures to the rest of the initially unknown isomers, based on fragment spectra, drift time, and RT information. Observed doubly charged species can further help in the elucidation of the one to three linked HMO branches and are found for HMOs equal to or beyond DP7. However, for HMOs exceeding the 2000 *m*/*z* range, spectra are generally characterized by presenting only doubly charged ions, which complicates the assignment of masses to the 1–6 linked HMO branches, for which singly charged ions are needed. Also, it is important to note that structural elucidation is not always possible due to the complexity and symmetry of some HMO structures, especially if the concentrations of particular compounds are too low to achieve sufficiently informative fragment ions.

**Figure 2:**
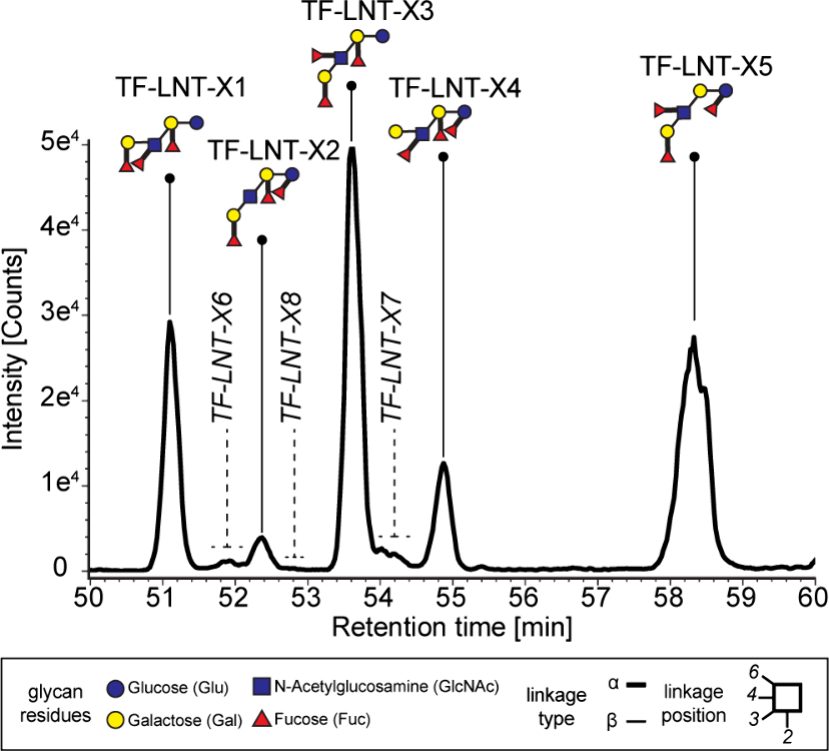
XIC of the [M+COOH]– adduct displaying the elution profile of the eight isomers detected for the trifucosylated-lacto-*N*-tetraoses. For isomers X1 to X5, their abundance is high enough to obtain sufficiently informative fragmentation spectra that enable structural elucidation. The proposed structural configuration for these novel HMO isomers is annotated over their corresponding isomer peak. The corresponding spectral annotations can be found in Figures S10–S14. Monosaccharide symbols and structural representations of HMOs were drawn in Illustrator according to the Consortium for Functional Glycomics81. Linkage type is represented by line thickness (α, thick line and β, thin line), while linkage position is represented by the orientation of the line.

For example, from eight trifucosyl-lacto-*N*-tetraose isomers (TF-LNTs) detected by accurate precursor ion mass readouts between 50 and 60 min RT in the long method, we could only propose a complete structure for TF-LNT-X1 to TF-LNT-X5 (Figure 2). These five isomers could be confidently annotated based on their IM and RT-deconvoluted fragmentation spectra (Figures S11–S15) despite being in the lower-abundance range. Although we do not yield all possible cross-ring fragments to determine linkage positions for all five characterized TF-LNTs (Figures S11–S15), the spectra confidently support fucose positioning. The glycosidic linkage types displayed on the proposed structures are also consolidated based on known rules for glucosyltransferase activity in HMO catabolism.^24,78^ As an exception from the known rules for fucose positioning at HMO backbones, we also could tentatively assign α1,2-linked fucoses to the internal galactose at the HMOs reducing end. In general, TF-LNTs were previously only found in human urine.^75^ The concentrations of the remaining TF-LNT-X6 to TF-LNT-X8 isomers were too low as to obtain sufficiently informative fragment ions, and no HMO structure could be proposed (Figure 2). Therefore, although our method can provide a steppingstone for structural determination of new HMO isomers, especially the wide diversity of complex and low-abundant HMOs calls for the use of additional complementary analytical methods for cross validation of tentative structural assignments.

A strategy similar to that described above for neutral HMOs can be used for the elucidation of acidic HMOs. However, this is less straightforward due to lower concentrations of many acidic HMOs present in HM, especially regarding the more complex compounds.^27^ The lower concentrations of these species lead to lower precursor intensities and, consequently, lower signal/noise ratios for the resulting fragment ions. Ultimately, this leads to less informative fragmentation spectra for the acidic HMOs. However, there is one feature that helps to determine that an HMO is of an acidic nature: Upon fragmentation, sialylated HMOs lead to a distinctive and prominent 290 *m*/*z* fragment, generated from the Neu5Ac moiety. Unfortunately, other fragments useful for structural elucidation of HMOs are an order of magnitude lower. While the higher concentrated acidic HMOs like 3′-SL, 6′-SL, LST a-c, and DSLNT can be determined using the long method as exemplified in Figure S16 for sialyllacto-*N*-tetraose a (LST a), the lower concentrated acidic HMOs would require prefractionation to remove the high-abundant HMOs prior to high-accuracy AIF CID fragmentation MS analysis. Finally, sulfated HMOs generate a distinct fragment ion of 97 *m*/*z*, corresponding to the loss of HSO_4_. However, complete elucidation of these compounds would need to be addressed in the future by a more dedicated approach^79^.

### Robust AIF LC-ESI-IM-MS for In-Depth Monitoring of Inter- and Intraindividual Variation in HMO Composition

**Figure 3:**
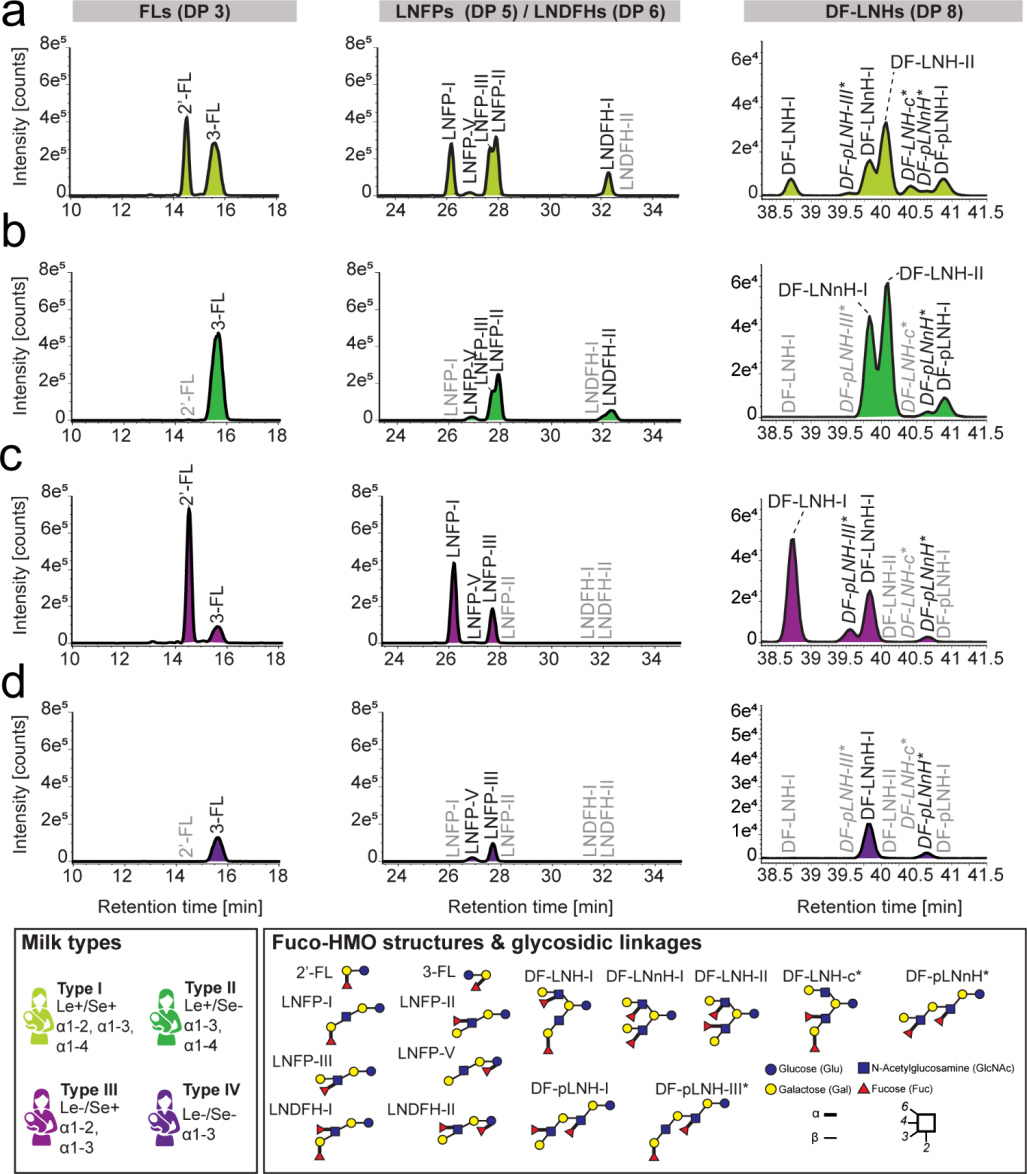
Visualization of the four (I–IV) different HM types using the XIC of the [M+COOH]– adducts measured with the short LC-ESI-IM-MS method. A selected array of fucosylated HMOs, covering known compounds of varying degrees of polymerization (DPs) and representative of milk types I–IV44. The selected HMOs include 2′-fucosyl-lactose isomers (DP = 3), four lacto-*N*-fucopentaose isomers (DP = 5), two lacto-*N*-difucohexaoses (DP = 6), and seven isomers of the difucosyl-lacto-*N*-hexaoses series (DP = 8). The elution profile of the selected isomers is displayed for analyzed milk derived from (a) a type-I donor, (b) a type-II donor, (c) a type-III donor, and (d) a type-IV donor. Monosaccharide symbols and structural representations of HMOs were drawn in Illustrator according to the Consortium for Functional Glycomic81. Linkage type is represented by line thickness (α, thick line and β, thin line), while linkage position is represented by the orientation of the line. An asterisk is used to highlight those structures, which were proposed based on informative fragmentation spectra, as derived from the analysis of the calibration reference sample using the long-method LC-ESI-IM-MS workflow (Figures S4- S10).

HM is a highly dynamic biofluid, where the overall composition, including HMOs, not only varies between mothers, but also throughout lactation for each individual mother.^5,6,24,25^ Analytical methods developed for advancing clinical research should be able to simultaneously measure a wide range of analytes while being robust enough to analyze hundreds of samples usually provided by HM cohort studies. Combining these two methodological properties should facilitate the accurate identification of key biological features with sound statistics. Since our method enables monitoring of hundreds of HMOs simultaneously, it does allow for precise characterization of very complex HMO profiles. Thereby, even inter- and intraindividual variations of complex and high molecular weight, yet low-abundant HMOs, can be followed. This depth of analysis may elicit yet unknown associations between these HMOs and early life maternal or infant factors. In a first application to HM samples, we challenged the capabilities of our AIF LC-ESI-IM-MS workflow with samples from donors of different milk types using a robust short method. It is well known that interindividual differences in HMO composition are greatly influenced by maternal genetic predispositions, particularly concerning the Le and Se genes. This results in four different major milk types with characteristic HMO profiles. Briefly, the Se and Le genes encode the FUT2 and the FUT3 enzymes, catalyzing the attachment of fucoses to HMO backbones via specific α1,2-glycosidic or α1,3/4-glycosidic linkages, respectively. Milk types I, II, III, and IV can therefore be distinguished by abundances and specific patterns of these fucosylated HMOs (Figure 3, legend). Analyzing different HMs from donors of milk types I–IV, we were able to monitor prototypic milk type-specific HMO profiles. Figure 3 displays prevalent milk type-specific fucosylated HMO structures, such as 2′-FL, 3-FL, LNFP I, LNFP II, LNDFH I, and LNDFH II.^44^ Also, less prevalent HMOs, such as the higher DP DF-LNHs, are visible and could be structurally characterized in HM samples based on fragmentation patterns (see Figures S4–S10). Indeed, the profiles also including these higher order HMOs were highly consistent with fucose linkage types as expected for the respective mother’s milk type (see samples from type-I–IV milks as displayed in Figure 3). This underscores the method’s reliability in deciphering biologically relevant structural HMO features. These results also showed that our method can accurately provide detailed information on underivatized lower or higher DP HMO isomers directly derived from mother’s milk.

**Figure 4:**
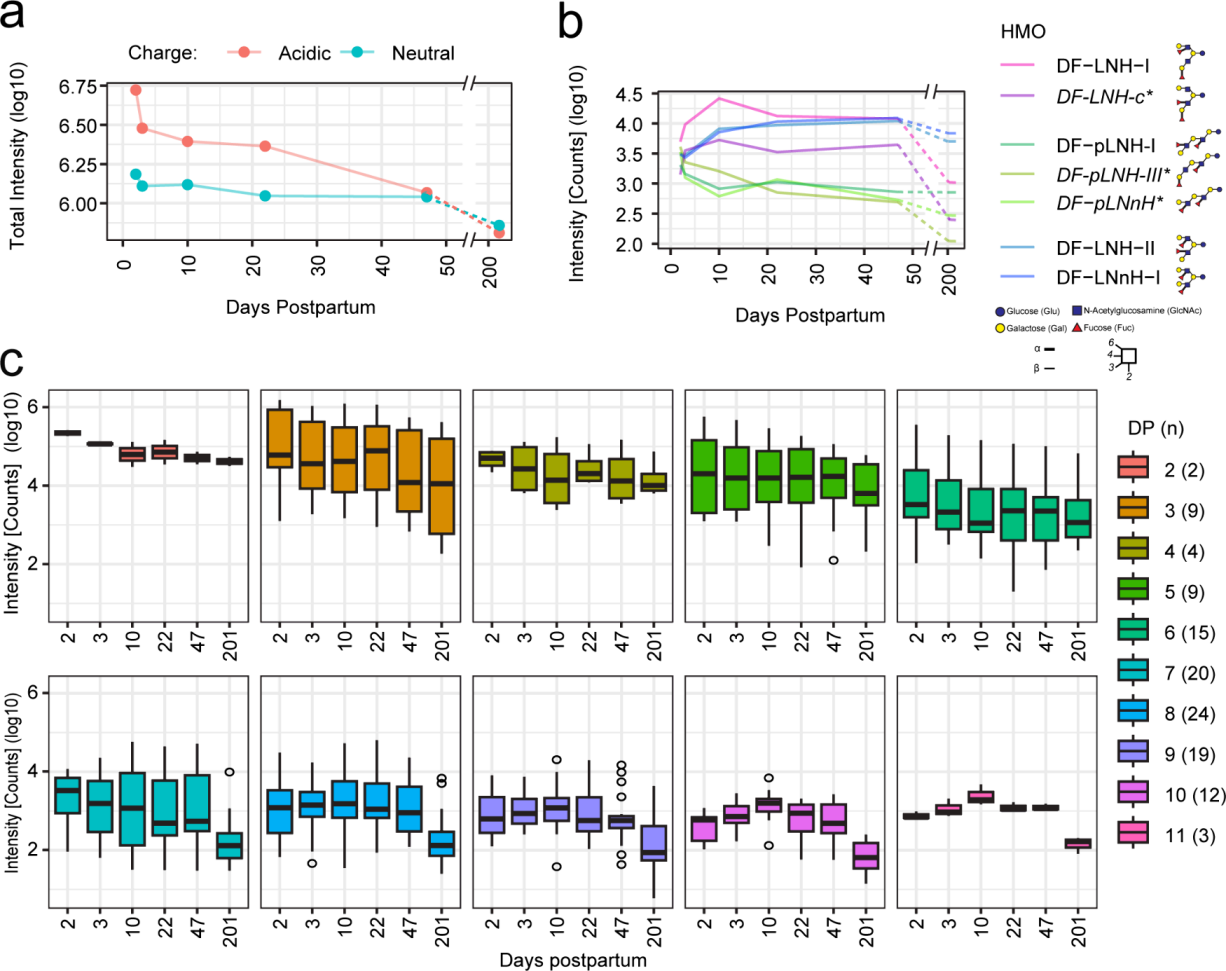
Characterization of milk from a type-I donor using the short-method AIF LC-ESI-IM-MS workflow reveals variation of HMO profiles over the course of lactation. To monitor changes over the course of lactation, milk collected at days 2, 3, 10, 22, 47, and 201 was analyzed. (a) The overall profile of neutral (blue) and acidic (red) HMOs showed a decreasing trend over the course of lactation. Acidic HMOs decreased eightfold and neutral HMOs decreased twofold over the course of lactation, as reflected in changes in the cumulative total intensity (*log10*) measured on day 2 and day 201. (b) The intensity (*log10*) of the seven isomers of the DF-LNH series is displayed over the course of lactation. Different isomer species vary following different patterns over time, peaking at varying time points. This behavior, which correlated with distinct structural HMO features, was observed for many other HMOs. The DF-LNH isomers whose structure was proposed based on informative fragmentation spectra, as derived from the analysis of the calibration reference sample using the long-method LC-ESI-IM-MS workflow (Figures S4–S10), are marked with an asterisk and italics font. Monosaccharide symbols and structural representations of HMOs were drawn in Illustrator according to the Consortium for Functional Glycomic81. Linkage type is represented by line thickness (α, thick line and β, thin line), while linkage position is represented by the orientation of the line. (c) A boxplot is shown summarizing the distribution of intensities (*log10*) measured across all HMOs within each DP across lactation. The box represents the first and third quartiles, and the midline represents the second quartile. The upper and lower whiskers extend from the hinge to the largest or smallest value within 1.5 times the interquartile range, respectively, and any points outside this interval are individually plotted and considered outliers. The number of HMOs (*n*) per DP is shown in the legend within the parentheses.

To further challenge the capabilities of this analytical approach, we intended to monitor the compositional changes of HMOs in milk from a type-I donor over different stages of lactation. Here, we chose to work with a milk type I donor because, based on their Le/Se genotypes and resulting fructosyltransferase profiles, they should provide the most complex array of HMOs (Figure 3A). Milk was collected at several time points from differing lactational stages: colostrum milk, days 2 and 3; transitional milk, days 10 and 22 and mature milk, days 47 and 201. These samples were analyzed with the short AIF LC-ESI-IM-MS workflow, obtaining a broad perspective of the dynamic changes in HMO profiles over lactation (Figure 4). In line with previous publications,^22,43^ we found the overall HMO content in milk to decline over the course of lactation, as reflected in a fivefold decrease in measured total HMO intensity for neutral and acidic compounds (Figure 4A). Intriguingly, not all HMOs showed the same pattern of variation throughout lactation. For example, even among isobaric isomers like the different DF-LNHs, three different patterns of variation were observed. These patterns in turn showed apparent correlations among DF-LNFHs sharing similar structural features, i.e., branching pattern (Figure 4B). The potential functional impact of these different branching patterns and the change of their levels over lactation for infants’ development is yet unknown and calls for further investigation.

When grouping HMOs per DP, shorter DP HMOs are most abundant in colostrum but decrease with milk maturity, potentially due to more pronounced prebiotic function, while higher DP HMOs remain relatively constant, peaking in transitional milk, and thus possibly suggesting another functional role, e.g., in infants’ development or in immune function (Figure 4C). Ranking HMOs by intensity and plotting S-curves at each time point reveals, again, complex individual variations, which could indicate diverse functions adapting to various infant needs (Figure S17A,B). Note that some HMO species are lost in more mature milk (Figure S17A,B, dots at zero intensity), highlighting the dynamic changes in HMO composition over different stages of lactation. Overall, our robust, short-method AIF LC-ESI-IM-MS workflow (Figure S1E,F) allows us to simultaneously monitor and quantify a large variety of HMOs in non-lactose depleted, nonderivatized HM derived from a single type-I milk donor. In total, up to 114 HMOs could be monitored from HM at day 2 of lactation, and while the number of HMOs detected decreased over the course of lactation, only up to 98 could still be detected at day 201 of lactation in the milk from the same donor. We caution that we tested our short method longitudinally with just one donor. Therefore, the interesting and novel findings described in Figure 4 and Figure S17 should be validated in larger cohorts, for which this method was specifically developed. However, if large sample sets are to be analyzed over a significant time span, assessing the need for normalization is advised, for which reference standards can be used.

## Conclusions

In this work, we introduce a robust AIF LC-ESI-IM-MS workflow to significantly advance HMO characterization in HM. We established a 4D analytical approach to reliably separate and refine peaks of coeluting HMO isomers, enabling detection of up to 203 HMOs in a reproducible way. Moreover, the implemented CID fragmentation strategy allows us to obtain high-accuracy fragmentation spectra, including valuable diagnostic ions, which aid in the structural elucidation of novel HMO isomeric structures for which standards are not commercially available. This highlights the potential of this method to better understand HM glycan compositions and structures. However, while new isomers can be confidently identified based on these four dimensions of analysis, it is important to acknowledge that the inherent complexity of HMO structures and their dynamic variations in concentrations may occasionally compromise the quality of spectra. Even with the methodological advances described here, this approach may still be insufficient to fully elucidate all possible HMO structures. In cases where structural determination is challenging, alternative techniques such as NMR,^80^ IMS-CID-IMS combined with cryogenic IR spectroscopy,^62,81^ or other approaches can be employed to overcome this limitation.

With our 4D AIF LC-ESI-IM-MS methodology, we simultaneously monitor abundance differences in 114 HMOs across real-world HM samples and stages of lactation between 2 and 201 days post partum for the first time. This may pave the way for result-driven future approaches that could help us to better understand the functional roles of HMOs in early life development and health. A further gain in knowledge might, for example, be achieved by coupling insights on simple to complex HMOs now amenable via 4D AIF LC-ESI-IM-MS with clinical data describing early life maternal and infants’ factors. Using this method may help to investigate in much more detail how dynamic changes in HM glycan profiles support infants’ health. This may in turn advance the development of tailored milk formulations best adapted to the infant’s needs and closest to breastmilk as a natural blueprint. Finally, this method may also be relevant to understand even better how mother’s milk types profoundly impact total HMO composition and may influence infant’s healthy development. We believe that with expanded HM cohorts and this powerful approach, we can accelerate the exploration of HMO benefits for early life and even adult life.

## Supporting Information Available

Optimization of stationary phase operation conditions for chromatographic separation of HMOs (S-2 to S-4); optimization of mass spectrometric parameters for robust HMO analysis (S-5 to S-7); fragmentation spectra of the DF-LNH isomers presented in Figures 1 and 3 (S-8 to S-14); fragmentation spectra of the novel TF-LNT isomers presented in Figure 2 (S-15 to S-19); fragmentation spectra of LST a (S-20); S-curves of HMO abundance over the course of lactation (S-21); commercial HMO standards used (Table S1, S-22) and measured HMOs per method (Tables S2, S-23) (PDF)
