# Supplementary material for: Robust and High-Resolution All-Ion Fragmentation LC-ESI-IM-MS Analysis for In-Depth Characterization or Profiling of Up to 200 Human Milk Oligosaccharides
Source: Anal Chem. 2025 Mar 6;97(10):5563–74. doi: 10.1021/acs.analchem.4c06081 (PMC11923967; doi:10.1021/acs.analchem.4c06081)
Supplement: Supplementary file 1 — ac4c06081_si_001.pdf [file ac4c06081_si_001.pdf]

SUPPORTING INFORMATION

Robust and high resolution all ion fragmentation LC-ESI-IM-MS analysis for in-depth characterization or profiling of up to 200 Human Milk Oligosaccharides (HMOs)

John Gonsalves[1], Julia Bauzá-Martinez[2], Bernd Stahl[1, 3], Kelly A. Dingess[1], Marko Mank[1*]

1 Danone Research & Innovation, Uppsalalaan 12, 3584 CT, Utrecht, The Netherlands.

2 Skid Visual Science, Castaños 106 1B, 08302, Mataró, Barcelona, Spain

3 Utrecht Institute for Pharmaceutical Sciences, Department of Chemical Biology & Drug Discovery, Utrecht University, 3584 CG Utrecht, The Netherlands .

TABLE OF CONTENTS

1. Optimization of stationary phase operation conditions for chromatographic separation of HMOs 2
 a) Optimal settings for PGC columns 2
 b) Optimal settings for HILIC-Amide columns 2
 c) Short and Long method gradients 3
 Figure S1 4
2. Optimization of mass spectrometric parameters for robust HMO analysis 5
 Figure S2 6
 Figure S3 7
3. Fragmentation spectra of the DF-LNH isomers presented in Figures 1 & 3 8
 Figure S4 8
 Figure S5 9
 Figure S6 10
 Figure S7 11
 Figure S8 12
 Figure S9 13
 Figure S10 14
4. Fragmentation spectra of the novel TF-LNT isomers presented in Figure 2 15
 Figure S12 16
 Figure S13 17
 Figure S14 18
 Figure S15 19
5. Fragmentation spectra of LST a 20
 Figure S15 20
6. S-curves of HMO abundance over the course of lactation 21
 Figure S17 21
7. Supplementary Tables 22
 Supplementary Table 1 22
 Supplementary Table 2 23
8. References 24

SUPPORTING INFORMATION

1. Optimization of stationary phase operation conditions for chromatographic separation of HMOs

In the work presented in this manuscript, we decided to utilize HILIC-Amide columns based on a thorough optimization and performance evaluation of this stationary phase. In fact, we contrasted the two most commonly used stationary phases in oligosaccharide analyses: PGC and HILIC-Amide columns[1], to select the more robust and better performing one as a steppingstone in our 4D LC-IM-MS method. More specifically, the stationary phases evaluated for the separation of HMOs using the reference sample were the Hypercarb PGC 2.1 mm x 150 mm internal diameter 3 µm particle size and 250 Å pore size (referred to as PGC, Thermo Fisher, 35003-152130) column and the Premier Glycan Amide 2.1 mm x 150 mm internal diameter 1.7 µm particle size and 130 Å pore size (referred to as HILIC-Amide, Waters, 186009976 and 186009524) column.

The PGC column can operate at pH = 0-14 and temperatures up to 105 ºC. Initially, the PGC column was operated at 75 ºC using the following mobile phases: eluent A consisted of 0.1 % FA in water and eluent B consisted of 0.1 % FA in ACN. However, using these conditions, and in line with previous evidence[2] we did not observe intended reduction to only one anomeric peak. At 75 ºC, a decrease in the stability of acidic HMOs was already seen and therefore higher temperatures were not evaluated. To try to minimize anomer peak splitting, we increased the pH of the mobile phase by substituting 0.1 % FA by 0.1% NH_4OH. While this approach helped switch to one anomeric species, it still required a temperature of 75 ºC to achieve anomer collapse into a single peak, and thus stability of acidic compounds was still compromised.

The HILIC-Amide column can operate in the pH = 3-7 range, and at temperatures of up to 70 ºC. In terms of pH, a high pH eluent is preferred for separation of oligosaccharides on HILIC-Amide columns for optimal resolution[3], as this helps minimize anomeric peak splitting. However, the high pH can reduce the RT stability of the acidic oligosaccharides over time in HILIC-Amide columns. We observed that at high pH, the RT of the acid oligosaccharides steadily decreased while the RT of neutral oligosaccharides remained relatively stable. To have a stable RT for all the oligomers, a slightly acidic pH, pH ≈ 4.4 (based on 100% water), was chosen for HILIC-Amide columns. At the selected pH, the acid oligosaccharides are mostly deprotonated, while the silanol groups of the stationary phase are mostly protonated, resulting in minimal repulsion between acid oligosaccharides and the stationary phase and thus, maximizing their stability inside the HILIC-Amide columns. To minimize anomer peak splitting of the oligosaccharides, a column temperature of 67.5 ºC was selected, and this temperature did not affect acid oligosaccharide stability on HILIC-Amide columns. To obtain optimal peak resolution and eluent stability, a 50 mM NH_4HCO_2 buffer was chosen. The concentration of the buffer provides the right balance between optimal peak width and optimal ionization efficiency of the oligosaccharides and gives sufficient stable retentions for up to 3-day runtime. Higher buffer concentrations lead to sharper peaks at the expense of ionization efficiency.

a) Optimal settings for PGC columns

The final selected conditions used for the PGC column experiments displayed in Figure S1 were the following: eluent A consisted of 0.1% NH_4OH in water and eluent B consisted of 0.1% NH_4OH in ACN. Both eluents were made by adding 1 mL Ammonium Hydroxide (28%) to 1 L of MilliQ water and ACN respectively. The column was operated at a temperature of 75 ºC. Under these conditions a similar level of acidic HMO degradation was found as that described Cao, C. *et al.*[2]. Nevertheless, the high temperature conditions were needed to reduce anomeric peak splitting. The short method operated as follows: samples were loaded for 1.5 min at 98% eluent A, at a flow rate of 0.4 mL/min, followed by a linear decrease to 70% A in 47 min, stabilization at 70% A for 1.5 min, followed by a steep decrease to 5% A in 0.5 min, a cleaning step at 5% A for 4 min, a steep increase to 98% A for 0.5 min and finally, re-equilibration at 98% A for 2 min. The long method operated as follows: samples were loaded for 1.5 min at 98% eluent A, at a flow rate of 0.4 mL/min, followed by a linear decrease to 70% A in 97 min, stabilization at 70% A for 1.5 min, followed by a steep decrease to 5% A in 0.5 min, a cleaning step at 5% A for 4 min, a steep increase to 98% A for 0.5 min and finally, re-equilibration at 98% A for 2 min.

b) Optimal settings for HILIC-Amide columns

The final selected conditions used in all the experiments displayed for the HILIC-Amide columns throughout the figures on this manuscript and throughout the supporting information are the following: eluent A consisted of 20% ACN with 50 mM ammonium formate (NH_4HCO_2) at pH ≈ 4.4, while eluent B consisted of 80% ACN with 50 mM NH_4HCO_2 at pH ≈ 4.4. Both eluents were made by adding 3.3 mL Ammonium Hydroxide (28%) and 2.2 mL FA (100%) to 1 L of 20% ACN and 80% ACN respectively. The columns were operated at a temperature of 67.5 ºC. Two Premier Glycan Amide columns were used in-series to increase separation power. Finally, two gradients were optimized for either in-

depth characterization of HMOs (**long method**, 108.5 min per sample) or higher-throughput analyses (**short method**, 59.5 min per sample).

c) Short and Long method gradients for HILIC-Amide Columns

The short method operated as follows: samples were loaded for 1.5 min at 2% eluent A, at a flow rate of 0.3 mL/min, followed by a linear increase to 35% A in 47 min, stabilization at 35% A for 2.5 min, followed by a steep increase to 95% A in 0.5 min at a flow rate of 0.225 mL/min, an isocratic wash at 95% A for 4 min, a steep decrease to 2% A for 0.5 min and finally, re-equilibration at 2% A for 2 min at a flow-rate of 0.350 mL/min.

The long method operated as follows: samples were loaded for 1.5 min at 2% eluent A, at a flow rate of 0.3 mL/min, followed by a linear increase to 32.5% A in 97 min, stabilization at 32.5% A for 1.5 min, followed by a steep increase to 95% A in 0.5 min at a flow rate of 0.225 mL/min, a cleaning step at 95% A for 4 min, a steep decrease to 2% A for 0.5 min and finally, re-equilibration at 2% A for 2 min at a flow-rate of 0.350 mL/min. For preparation of the buffers 2.2 mL FA (Fisher Scientific, A117-2AMP) and 3.3 ml NH_4OH were added to 1 L of 20% ACN (Eluent A) and 80% ACN (Eluent B).

The optimal settings for each column were compared, and the results are displayed in **Figure S1** in this section. Compared to PGC columns, the HILIC-Amide column separated HMOs in a more predictable way, with HMOs eluting in order of increasing DP (**Figure S1A**) as recently reported[4]. Using PGC, different DPs did not correlate as clearly with the elution profile (**Figure S1B**). This result could be attributed to the different mode of interaction for both stationary phases. Since PGC interactions are based on Londen-type dispersive interaction, size and shape as well as the polar retention effect on graphite[5], they will tend to separate oligosaccharides based on structural composition and electron ion pair donating/accepting interactions. Differently, the hydrophilic partitioning, hydrogen bonding and electrostatic interaction characteristics of HILIC-Amide columns will promote stronger interactions the more hydrophilic the HMOs structures are. In general, hydrophilic properties of HMOs do increase with their DP which, in turn, might explain DP-dependent separation of HMOs using HILIC-Amide. Interestingly, the charge of the HMOs did not have a strong influence on the elution pattern with HILIC-Amide. In addition, the acidic HMO fraction eluted in DP-dependent order, but with a slight shift towards higher RT as compared to their non-acidic counterparts of the same DP.

For PGC, under the selected optimal operation conditions, the elution pattern of acidic HMOs was more erratic (**Figure S1A-B**). On the other hand, the PGC stationary phase stretched the elution of several isobaric HMO isomers over a longer RT range which resulted in better overall isomer separation (**Figure S1A-B**) compared to HILIC-Amide columns. As a result, separation performance of both columns was comparable up to DP-8 for neutral compounds (**Figure S1C, Supplementary Table 2**), but PGC was superior in resolution of some higher order HMOs such as Difucosyllacto-N-Octaose (DF-LNO). This broadened RT range for higher order isobaric HMO isomers translated into better isomer separation. This is an advantage in the analysis of such complex higher order HMOs as the number of isomers increases at higher DPs. Based on RT alone, HILIC-Amide columns could distinguish fewer isomers (**Supplementary Table 2**). However, the HILIC-Amide column was more beneficial for separation of acidic HMOs as mentioned above (**Supplementary Table 2**). Overall, our data show that the number of detectable HMO isomers varied between both stationary phases depending on the HMO physicochemical properties which influenced their elution patterns (**Supplementary Table 2**). Finally, 197 and 203 HMOs in the DP range 2-13 could be detected in the calibration reference samples following LC separation with either PGC or HILIC-Amide columns respectively.

Another important parameter that would drive the choice of stationary phase is compound stability over time within the column of choice. Overall, stability over time is a key parameter to achieve accurate characterization and quantitation of milk HMO profiles, especially for longitudinal, higher-throughput studies. To determine the stability of HMOs in either HILIC-Amide or PGC columns, we performed 48 repeated injections of a standard milk sample using the short method. Each stability series consists of a total 67 injections, including the 48 injection replicates, standards, blanks, and system conditioning, leading to about 67 hours of runtime, which is equal to the time needed for a batch of regular samples. Our results showed that HILIC-Amide columns displayed extraordinary RT-stability (±0.1 min) and signal-stability over time, with 64% of HMOs displaying CVs < 10% (**Figure S1F**), including a large portion of more abundant HMOs, both acidic and neutral, displaying CVs < 5% (**Figure S1F**). As expected, HMOs with higher variabilities were consistently in the lower abundance range (data not shown). On the contrary, PGC columns displayed less stability over the same number of injections (**Figure S1G**) which could be attributed to the effect of LC conditions required to ensure collapse of anomeric peaks into one single signal. The final optimal LC conditions also rendered a better signal stability over time on HILIC-Amide columns rather than on PGC columns (**Figure S1 F, G**). The benefits of HILIC-Amide stationary phase in terms of predictability, narrower RT ranges, repeatability and quantitative stability made it the better choice for comprehensive HMO analyses. Consequently, only HILIC-Amide was subsequently applied as stationary phase to characterize HMOs profiles in selected HM samples.

SUPPORTING INFORMATION

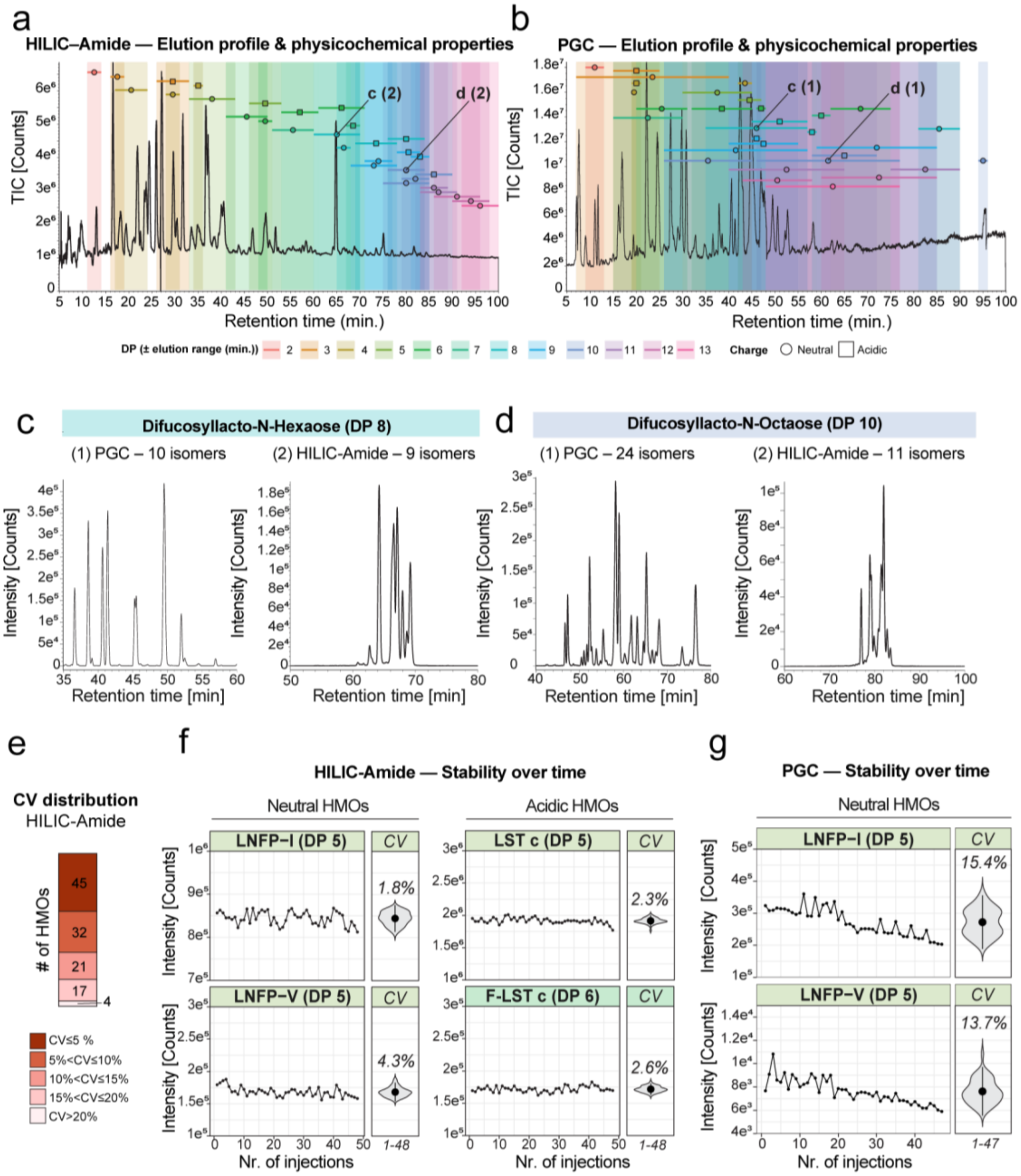

Figure S1. HILIC-Amide column stationary phase offers enhanced predictability, reproducibility, and stability over time for HMO separation. (**a**) Elution profile over a 108.5 min run for calibration reference sample loaded into a HILIC-Amide columns and (**b**) elution profile on a porous graphitized carbon (PGC) column. Eluting HMOs are highlighted according to two physicochemical properties, acidity or neutrality of the HMO and degree of polymerization (DP). The retention time (RT) range where each isomer series elutes is highlighted with a DP-colored line. At RT mid-point, a circle represents neutral HMOs and a square represents acidic HMOs. (**c**) Elution profile of Difucosyllacto-N-Hexaose on a (1) PGC column, where 10 isomers can be seen and on a (2) HILIC-Amide column, where 9 isomers can be seen. (**d**) Elution profile of Difucosyllacto-N-Octaose on a (1) PGC column, where 24 isomers can be seen and on a (2) HILIC-Amide column, where 11 isomers can be seen. (**e**) Number of HMOs per slice of CV for the HILIC-Amide 48 injections series. (**f**) Peak stability over time for the neutral HMOs LNFP-I and LNFP-V, as well as for the acidic HMOs Sialyllacto-N-tetraose c (LST c) and Fucosylsialyllacto-N-neotetraose c (F-LSTc) on a HILIC-Amide column over 48 consecutive injections of SRM1953 milk samples. A violin plot summarizing the 48 injections is displayed on the right, and the observed coefficient of variation (CV) is annotated on top. (**g**) Peak stability over time for the neutral HMOs Lacto-N-Fucopentaose I and V (LNFP-I and -V) on a PGC column over 48 consecutive injections of SRM1953 milk samples. A violin plot summarizing the 47 injections is displayed on the right, and the observed CV is annotated on top. For all plots, DP is annotated following the color code displayed on legend for panels **a-b.**

2. Optimization of mass spectrometric parameters for robust HMO analysis

Human milk contains both acidic and neutral HMOs, posing one more analytical challenge for AIF LC-ESI-IM-MS characterization. In this regard, at the mass spectrometer level, optimization of ionization, adduct selection and fragmentation are paramount to ensure accurate identification and quantitation of the entire range of acidic and neutral HMOs present in human milk.

We first optimized source parameters for adequate ionization and transmission of both acidic and neutral HMOs using a milk standard sample (**Figure S2A**). Acidic HMOs are more easily ionizable in the negative ESI mode, generating mostly [M-H]- ions, and suffering negligible in-source fragmentation. On the contrary, neutral HMOs are harder to ionize and are more prone to in-source fragmentation. For neutrals, besides deprotonated precursor ions ([M-H]-) (**Figure S3**, green), formate adducts ([M+HCOO]-) (**Figure S3**, gray) are frequently generated through interaction with ammonium formate (NH_4HCO_2) present in the mobile phase. In fact, the neutral HMO-specific formate addduct ions are advantageous, since they can serve as a selection tool to distinguish between coeluting acidic and neutral HMOs (**Figure S3**). Given that sialylated and difucosylated HMOs differ by ~1Da, the second isotope can interfere (note the corresponding [M-H]-, green-shaded ions for LSTc and LNDFH-I in **Figure S3C-D**). However, apart from the informative formate ions, other in-source generated fragment ions, such as [M-117]- (corresponding to a loss of glucose at the reducing end +HCOO, such as for LNFP-I in Figure S2A) and [M-263]- (corresponding to the loss of glucose and fucose at the reducing end glucose +HCOO, such as for LNFP-V in **Figure S3B**) are frequently produced and should be minimized to achieve more sensitivity and reliable quantitation. From various parameters tested, tuning capillary voltage, desolvation temperature and desolvation gas flow was sufficient to maximize ionization efficiency while minimizing in-source fragmentation. A compromise was found at 0.5 kV capillary voltage, 550ºC desolvation temperature and1000 L/h gas flow (**Figure S2A**, red) when using the HILIC-Amide column. The source settings were the same for analysis done with the PGC column except for the capillary voltage which was optimal at 1.25 kV. It must be noted that the [M-H]- ions were the predominant species due to the absence of formic acid in the eluent.

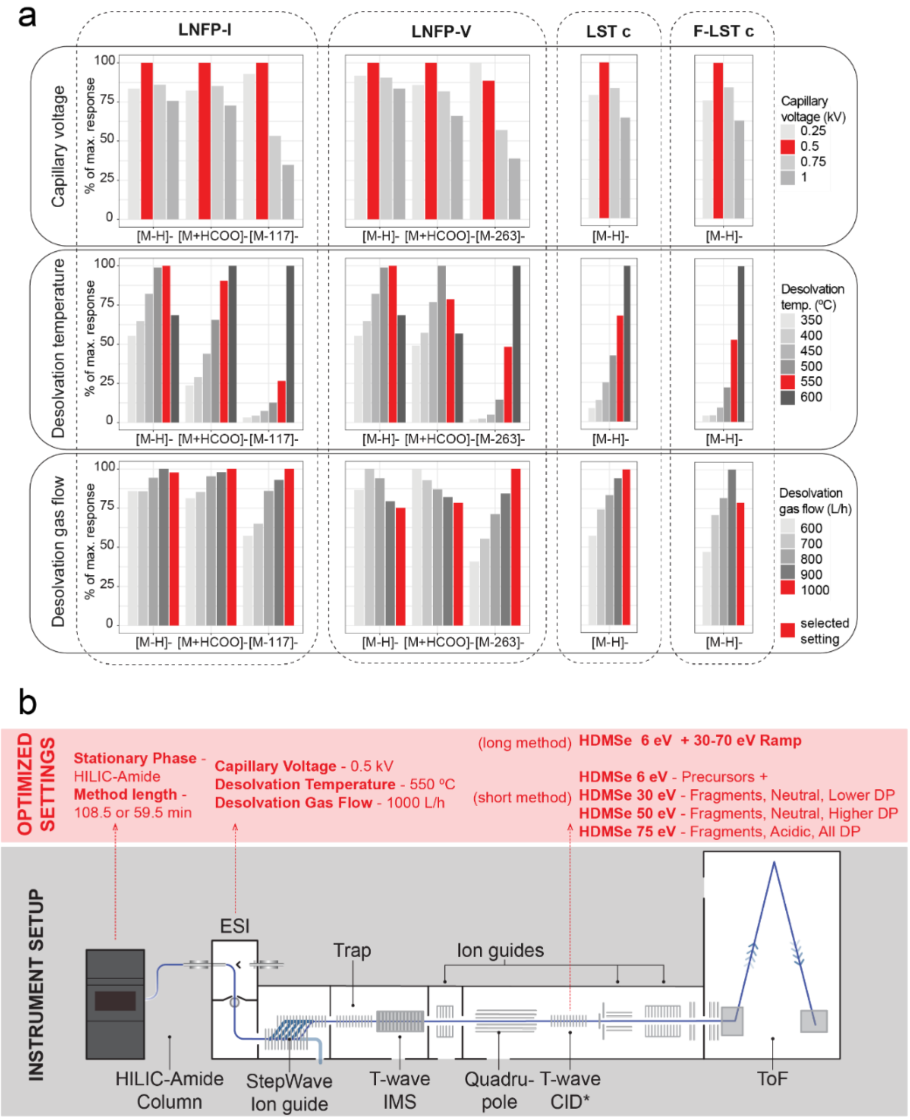

Figure S2. Optimization of relevant settings to achieve optimal ionization, transmission, and fragmentation of neutral and acidic HMOs on an LC-ESI-IMS-MS instrumental setup. (**a**) Capillary voltage, Desolvation temperature and Desolvation gas flow were modulated to select the settings that maximize ionization efficiency while minimizing in-source fragmentation. The performance of the different settings tested is exemplified using 2 neutral HMOs (LNFP I and V) and 2 acidic HMOs (LST c and F-LST c), for which the % of the maximum response measured is displayed. The selected settings are highlighted in red. (**b**) The final parameters used in the short and long method AIF LC-ESI-IMS-MS workflow throughout the manuscript are displayed over the diagram of the system used, *i.e.*, an Acquity UPLC I class plus coupled to a Vion IMS Qtof MS.

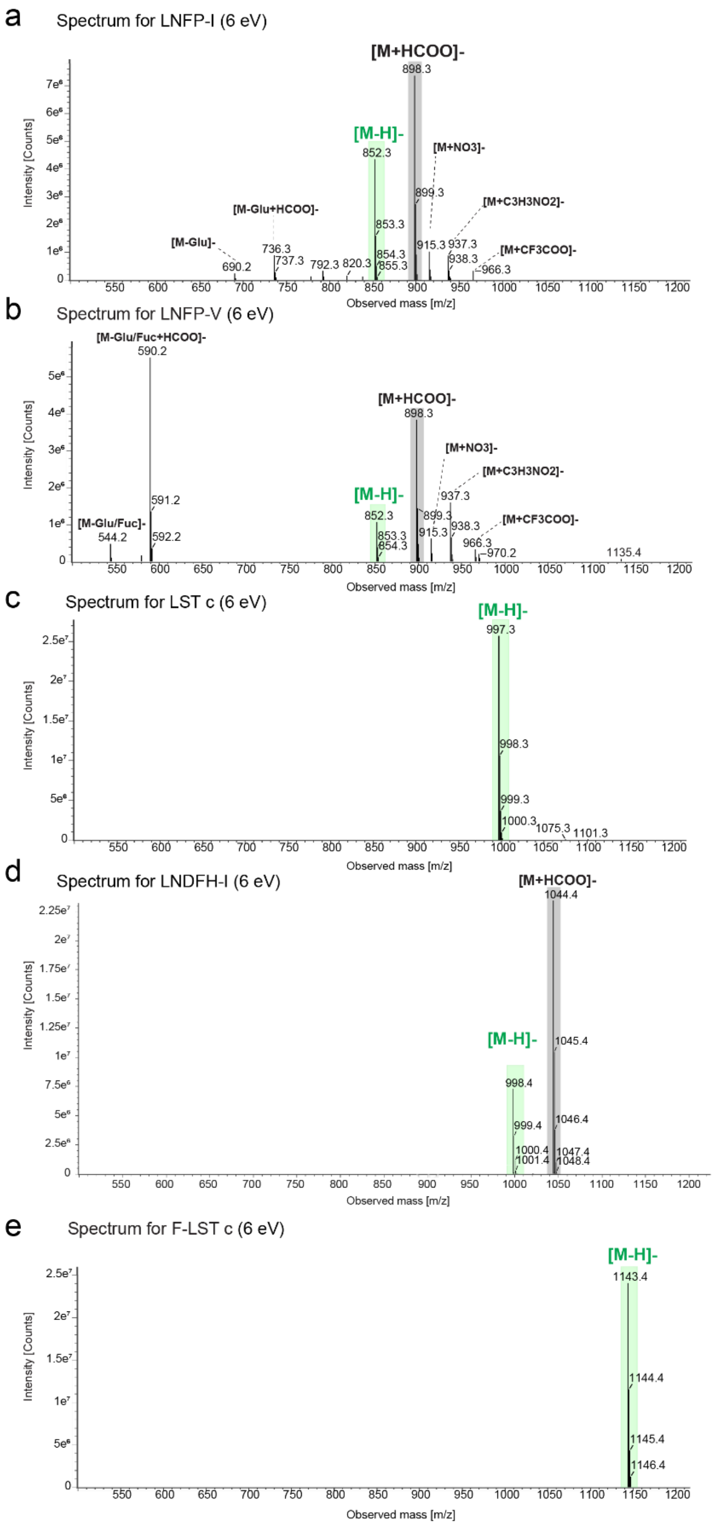

Figure S3. Precursor ion scans (6eV HDMSe) for selected HMOs. (**a**) Spectrum for LNFP-I. (**b**) Spectrum for LNFP-V. (**c**) Spectrum for LST c, and (**d**) Spectrum for LNDFH-I, showing 1Da mass difference to that of LST c. (**e**) Spectrum for F-LST c. The neutral HMOs LNFP-I and LNFP-V together with the acidic HMOs LST c and F-LST c were used for parameter optimization and stability tests displayed in **Figure S1** and **Figure S2**. Retention- and drift time deconvoluted fragmentation spectra are obtained using the optimal settings selected for negative-mode ESI. Deprotonated precursor [M-H]- is shaded in green, the frequently found formate adduct [M+HCOO]- is shaded in gray, and other adducts / in-source generated fragments are annotated where relevant.

3. Fragmentation spectra of the DF-LNH isomers presented in Figures 1 & 3

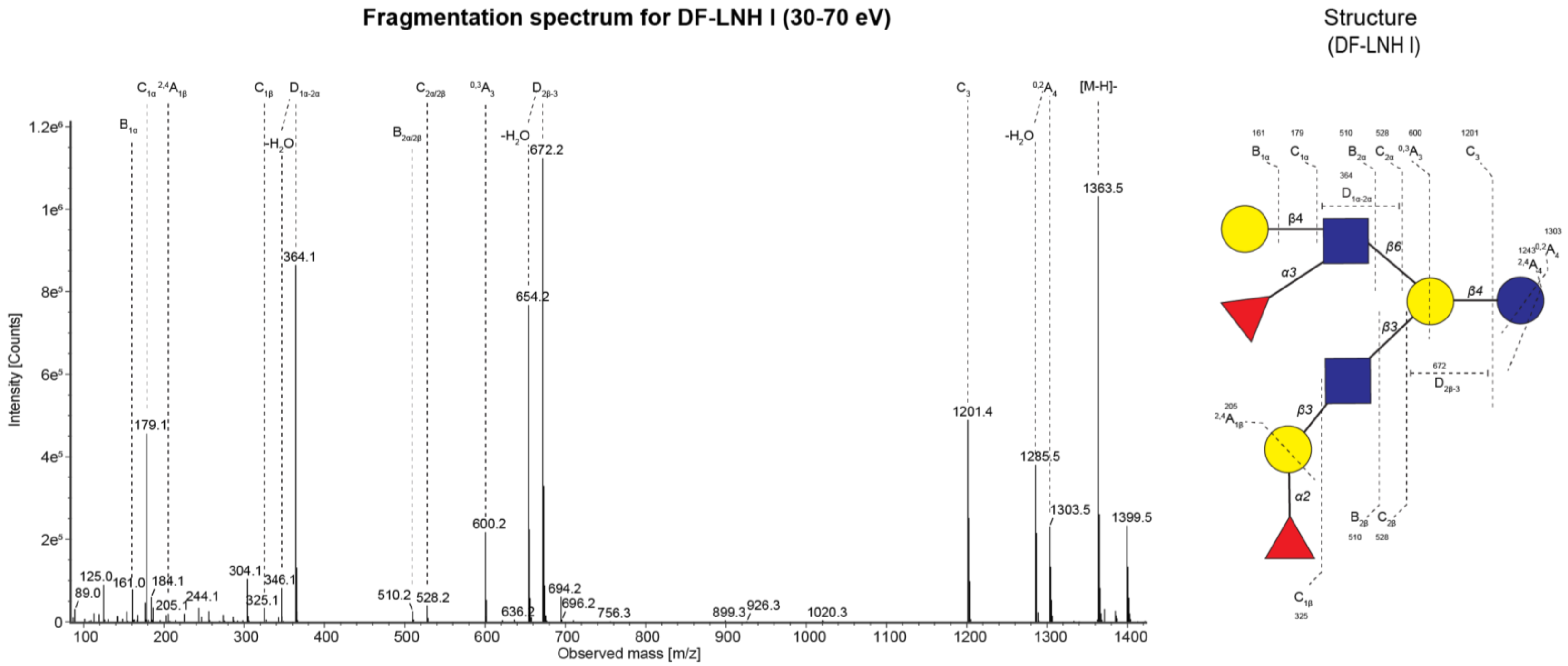

Figure S4. Retention- and drift time deconvoluted fragmentation spectrum for DF-LNH-I. Spectrum was obtained analyzing the calibration reference sample using the long method LC-ESI-IMS-MS workflow. CID fragmentation energy was ramped from 30 to 70 eV for all ions fragmentation (AIF). In the spectrum, A, B, and C fragments are annotated over the peaks following Domon & Costello's nomenclature[6], while D-fragments, resulting from a consecutive fragmentation into a C fragment and Z fragment, are annotated as proposed by Chai, Piskarev & Lawson[7,8]. Fragment ions that were not measured in the spectrum upon D-type fragmentation are annotated in gray. When relevant, water losses (-H_2O) are annotated next to their corresponding fragment ion by a dotted line. The known structure is annotated to the right of the spectrum, together with the fragment ions observed at each monosaccharide residue. Monosaccharide symbols and structural representations of HMOs were drawn in Illustrator according to the Consortium for Functional Glycomics[9]. Linkage type (α or β) together with linkage position is annotated directly on the structure.

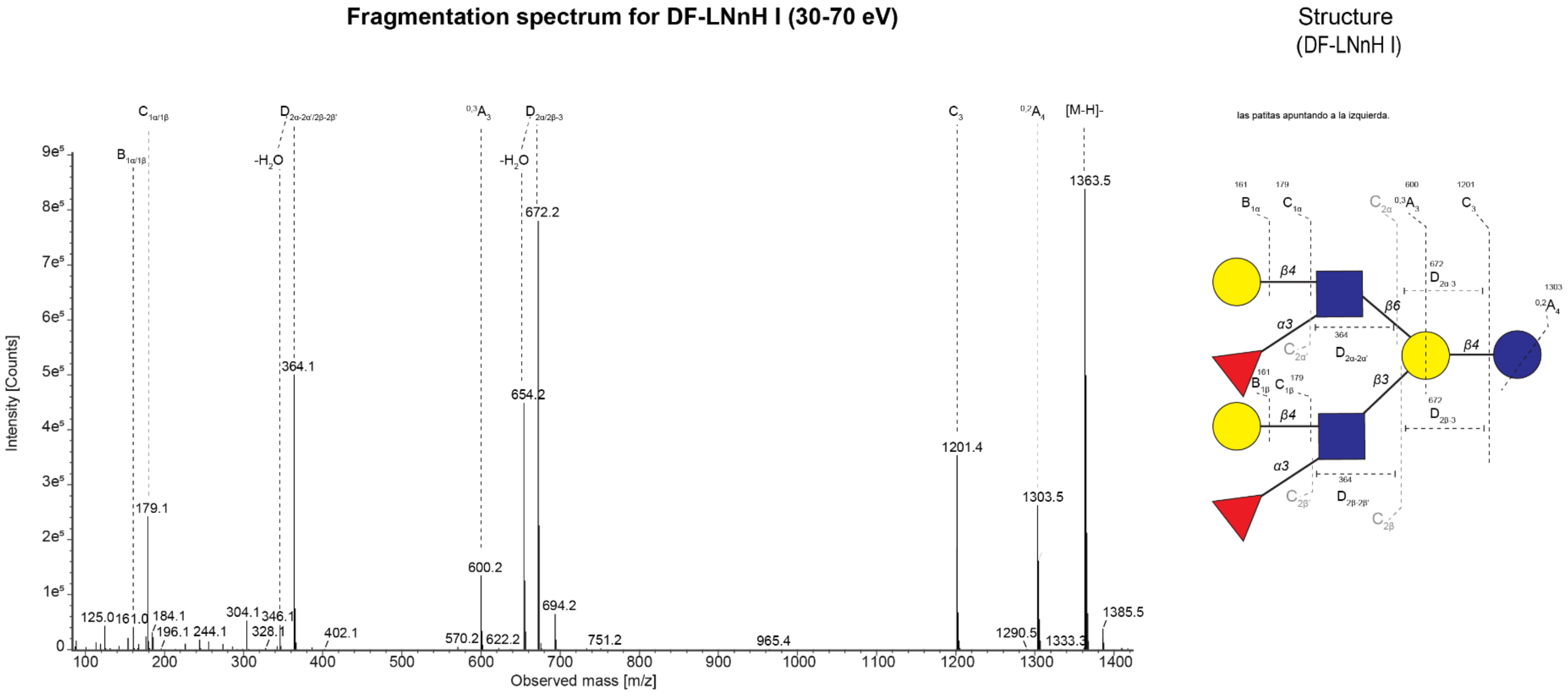

Figure S5. Retention- and drift time deconvoluted fragmentation spectrum for DF-LNnH-I. Spectrum was obtained analyzing the calibration reference sample using the long method LC-ESI-IMS-MS workflow. CID fragmentation energy was ramped from 30 to 70 eV for all ions fragmentation (AIF). In the spectrum, A, B, and C fragments are annotated over the peaks following Domon & Costello's nomenclature[6], while D-fragments, resulting from a consecutive fragmentation into a C fragment and Z fragment, are annotated as proposed by Chai, Piskarev & Lawson[7,8]. Fragment ions not measured in the spectrum on D-type fragmentation are annotated in gray. When relevant, water losses (-H_2O) are annotated next to their corresponding fragment ion by a dotted line. The known structure is annotated to the right of the spectrum, together with the fragment ions observed at each monosaccharide residue. Monosaccharide symbols and structural representations of HMOs were drawn in Illustrator according to the Consortium for Functional Glycomics[9] . Linkage type (α or β) together with linkage position is annotated directly on the structure.

SUPPORTING INFORMATION

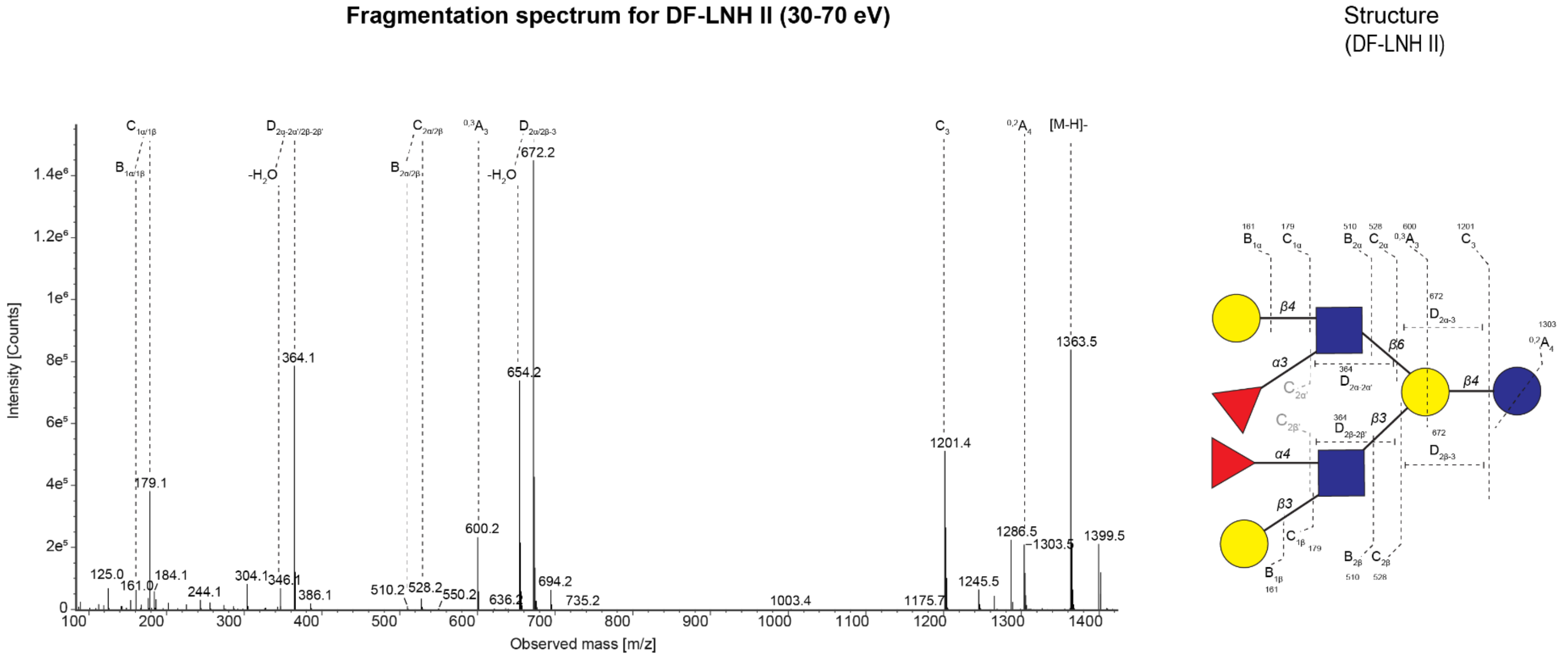

Figure S6. Retention- and drift time deconvoluted fragmentation spectrum for DF-LNH-II. Spectrum was obtained analyzing the calibration reference sample using the long method LC-ESI-IMS-MS workflow. CID fragmentation energy was ramped from 30 to 70 eV for all ions fragmentation (AIF). In the spectrum, A, B, and C fragments are annotated over the peaks following Domon & Costello's nomenclature[6], while D-fragments, resulting from a consecutive fragmentation into a C fragment and Z fragment, are annotated as proposed by Chai, Piskarev & Lawson[7,8]. Fragment ions not measured in the spectrum on D-type fragmentation are annotated in gray. When relevant, water losses ($-H_2O$) are annotated next to their corresponding fragment ion by a dotted line. The known structure is annotated to the right of the spectrum, together with the fragment ions observed at each monosaccharide residue. Monosaccharide symbols and structural representations of HMOs were drawn in Illustrator according to the Consortium for Functional Glycomics[9]. Linkage type (α or β) together with linkage position is annotated directly on the structure.

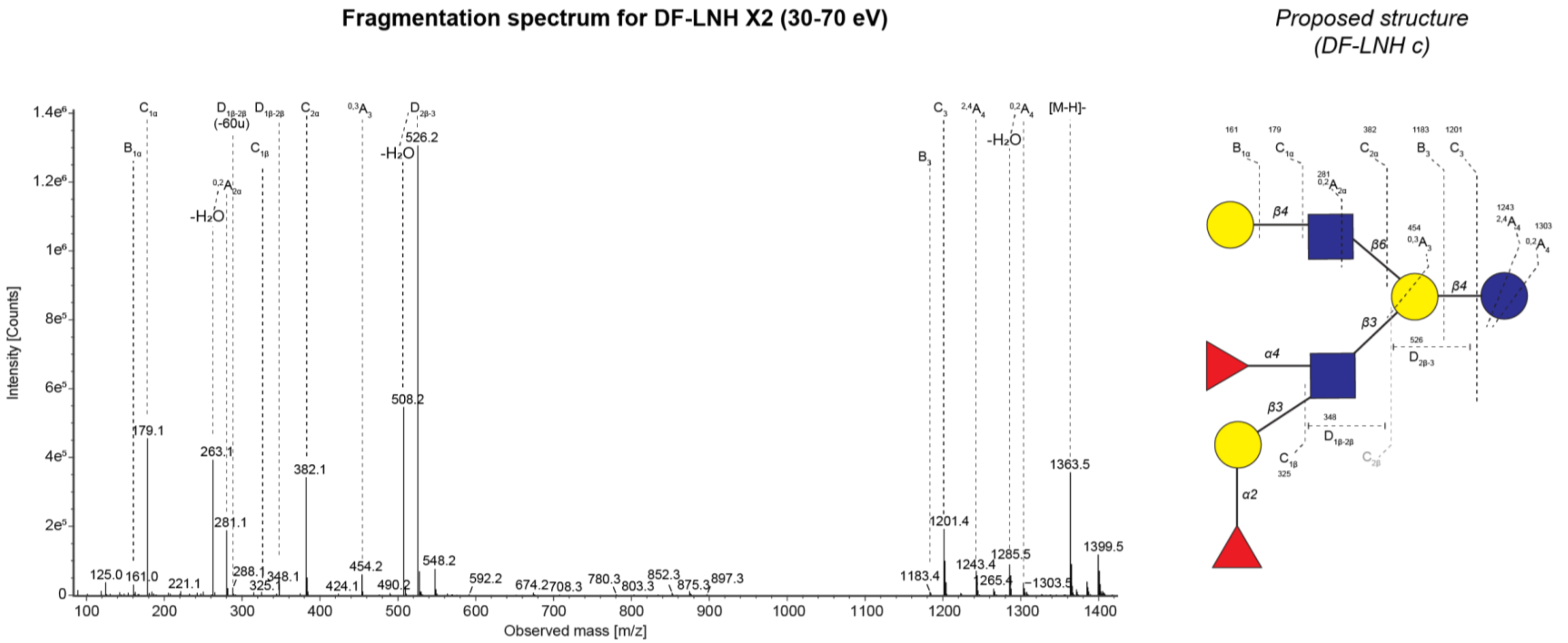

Figure S7. Retention- and drift time deconvoluted fragmentation spectrum for DF-LNH-X2. Spectrum was obtained analyzing the calibration reference sample using the long method LC-ESI-IMS-MS workflow. CID fragmentation energy was ramped from 30 to 70 eV for all ions fragmentation (AIF). In the spectrum, A, B, and C fragments are annotated over the peaks following Domon & Costello's nomenclature[6,] while D-fragments, resulting from a consecutive fragmentation into a C fragment and Z fragment, are annotated as proposed by Chai, Piskarev & Lawson[7,8.] Fragment ions not measured in the spectrum on D-type fragmentation are annotated in gray. When relevant, water losses (-H_2O) are annotated next to their corresponding fragment ion by a dotted line. The structure of this isomer was originally unknown (isomer X2 of the DF-LNH series), but based on the spectral information a structure can be predicted and it is annotated to the right of the spectrum, together with the fragment ions observed at each monosaccharide residue. The proposed structure coincides with the previously described DF-LNH c compound[10.] Monosaccharide symbols and structural representations of HMOs were drawn in Illustrator according to the Consortium for Functional Glycomics[9]. Linkage type (α or β) together with linkage position is annotated directly on the structure.

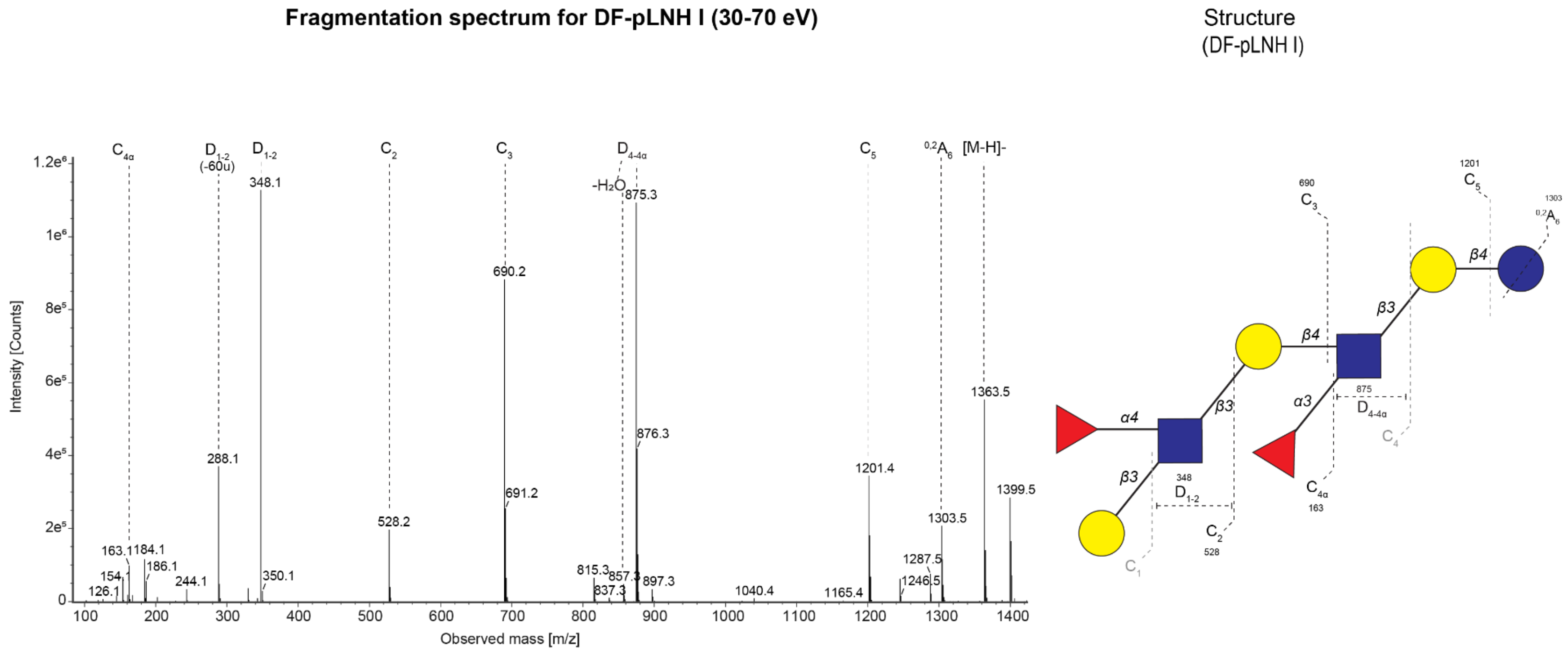

Figure S8. Retention- and drift time deconvoluted fragmentation spectrum for DF-pLNH-I. Spectrum was obtained analyzing the calibration reference sample using the long method LC-ESI-IMS-MS workflow. CID fragmentation energy was ramped from 30 to 70 eV for all ions fragmentation (AIF). In the spectrum, A, B, and C fragments are annotated over the peaks following Domon & Costello's nomenclature[6], while D-fragments, resulting from a consecutive fragmentation into a C fragment and Z fragment, are annotated as proposed by Chai, Piskarev & Lawson[7,8]. Fragment ions not measured in the spectrum on D-type fragmentation are annotated in gray. When relevant, water losses (-H_2O) are annotated next to their corresponding fragment ion by a dotted line. The known structure is annotated to the right of the spectrum, together with the fragment ions observed at each monosaccharide residue. Monosaccharide symbols and structural representations of HMOs were drawn in Illustrator according to the Consortium for Functional Glycomics[9]. Linkage type (α or β) together with linkage position is annotated directly on the structure.

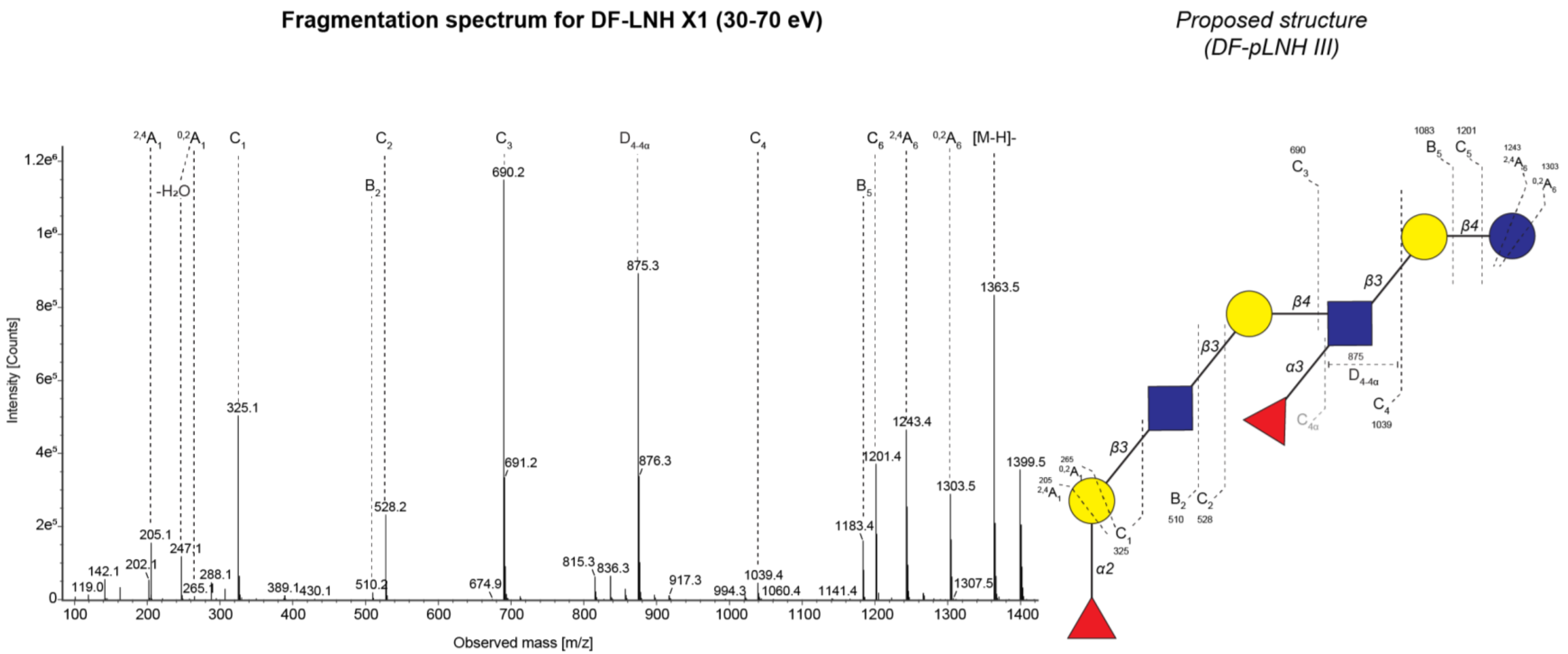

Figure S9. Retention- and drift time deconvoluted fragmentation spectrum for DF-LNH-X1. Spectrum was obtained analyzing the calibration reference sample upon long-gradient LC-ESI-IMS-AIF analysis. CID fragmentation energy was ramped from 30 to 70 eV for all ions fragmentation (AIF). In the spectrum, A, B, and C fragments are annotated over the peaks following Domon & Costello's nomenclature[6], while D-fragments, resulting from a consecutive fragmentation into a C fragment and Z fragment, are annotated as proposed by Chai, Piskarev & Lawson[7,8]. Fragment ions not measured in the spectrum on D-type fragmentation are annotated in gray. When relevant, water losses (-H_2O) are annotated next to their corresponding fragment ion by a dotted line. The structure of this isomer was originally unknown (isomer X1 of the DF-LNH series) but based on the spectral information a structure can be predicted and it is annotated to the right of the spectrum, together with the fragment ions observed at each monosaccharide residue. The proposed structure coincides with the previously described DF-pLNH III compound[11]. Monosaccharide symbols and structural representations of HMOs were drawn in Illustrator according to the Consortium for Functional Glycomics[9]. Linkage type (α or β) together with linkage position is annotated directly on the structure.

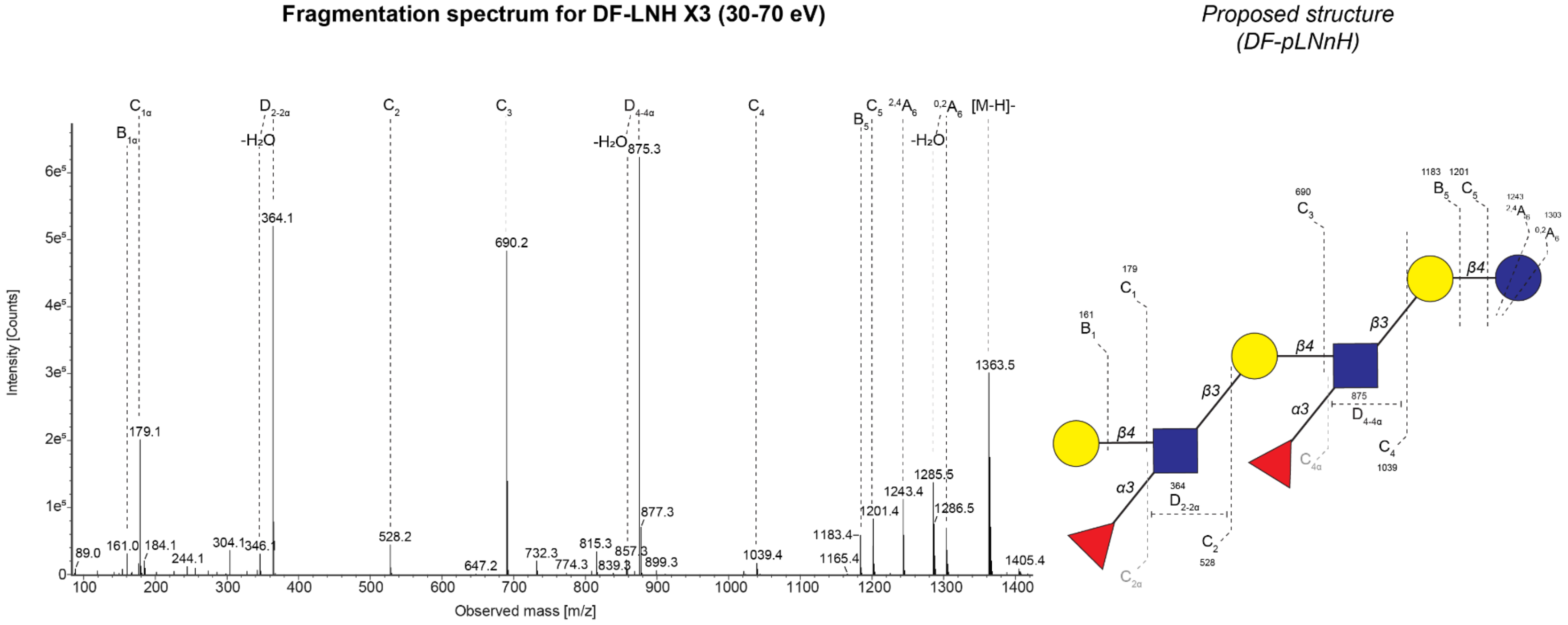

Figure S10. Retention- and drift time deconvoluted fragmentation spectrum for DF-LNH-X3. Spectrum was obtained analyzing the calibration reference sample using the long method LC-ESI-IMS-MS workflow. CID fragmentation energy was ramped from 30 to 70 eV for all ions fragmentation (AIF). In the spectrum, A, B, and C fragments are annotated over the peaks following Domon & Costello's nomenclature[6], while D-fragments, resulting from a consecutive fragmentation into a C fragment and Z fragment, are annotated as proposed by Chai, Piskarev & Lawson[7,8]. Fragment ions not measured in the spectrum on D-type fragmentation are annotated in gray. When relevant, water losses (-H_2O) are annotated next to their corresponding fragment ion by a dotted line. The structure of this isomer was originally unknown (isomer X3 of the DF-LNH series) but based on the spectral information a structure can be predicted and it is annotated to the right of the spectrum, together with the fragment ions observed at each monosaccharide residue. The proposed structure coincides with the previously described DF-pLNnH compound[10]. Monosaccharide symbols and structural representations of HMOs were drawn in Illustrator according to the Consortium for Functional Glycomics[9]. Linkage type (α or β) together with linkage position is annotated directly on the structure.

4. Fragmentation spectra of the novel TF-LNT isomers presented in Figure 2

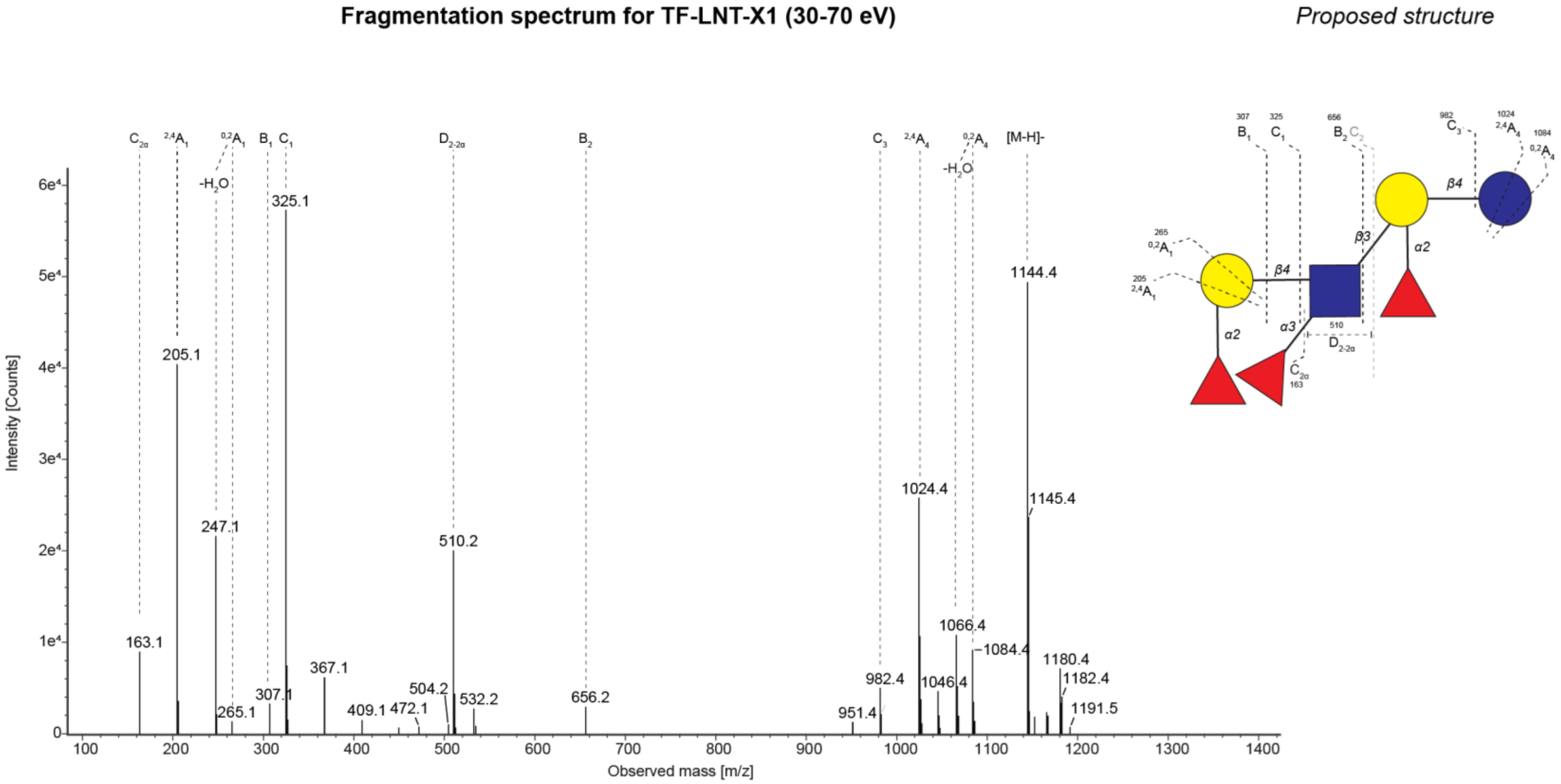

Figure S11. Retention- and drift time deconvoluted fragmentation spectrum for TF-LNT-X1. Spectrum was obtained analyzing the calibration reference sample using the long method LC-ESI-IMS-MS workflow. CID fragmentation energy was ramped from 30 to 70 eV for all ions fragmentation (AIF). In the spectrum, A, B, and C fragments are annotated over the peaks following Domon & Costello's nomenclature[6], while D-fragments, resulting from a consecutive fragmentation into a C fragment and Z fragment, are annotated as proposed by Chai, Piskarev & Lawson[7,8]. Fragment ions not measured in the spectrum on D-type fragmentation are annotated in gray. When relevant, water losses (-H_2O) are annotated next to their corresponding fragment ion by a dotted line. The structure of this isomer was originally unknown (isomer X1 of the TF-LNTseries) but based on the spectral information a structure can be predicted and it is annotated to the right of the spectrum, together with the fragment ions observed at each monosaccharide residue. Monosaccharide symbols and structural representations of HMOs were drawn in Illustrator according to the Consortium for Functional Glycomics[9]. Linkage type (α or β) together with linkage position is annotated directly on the structure.

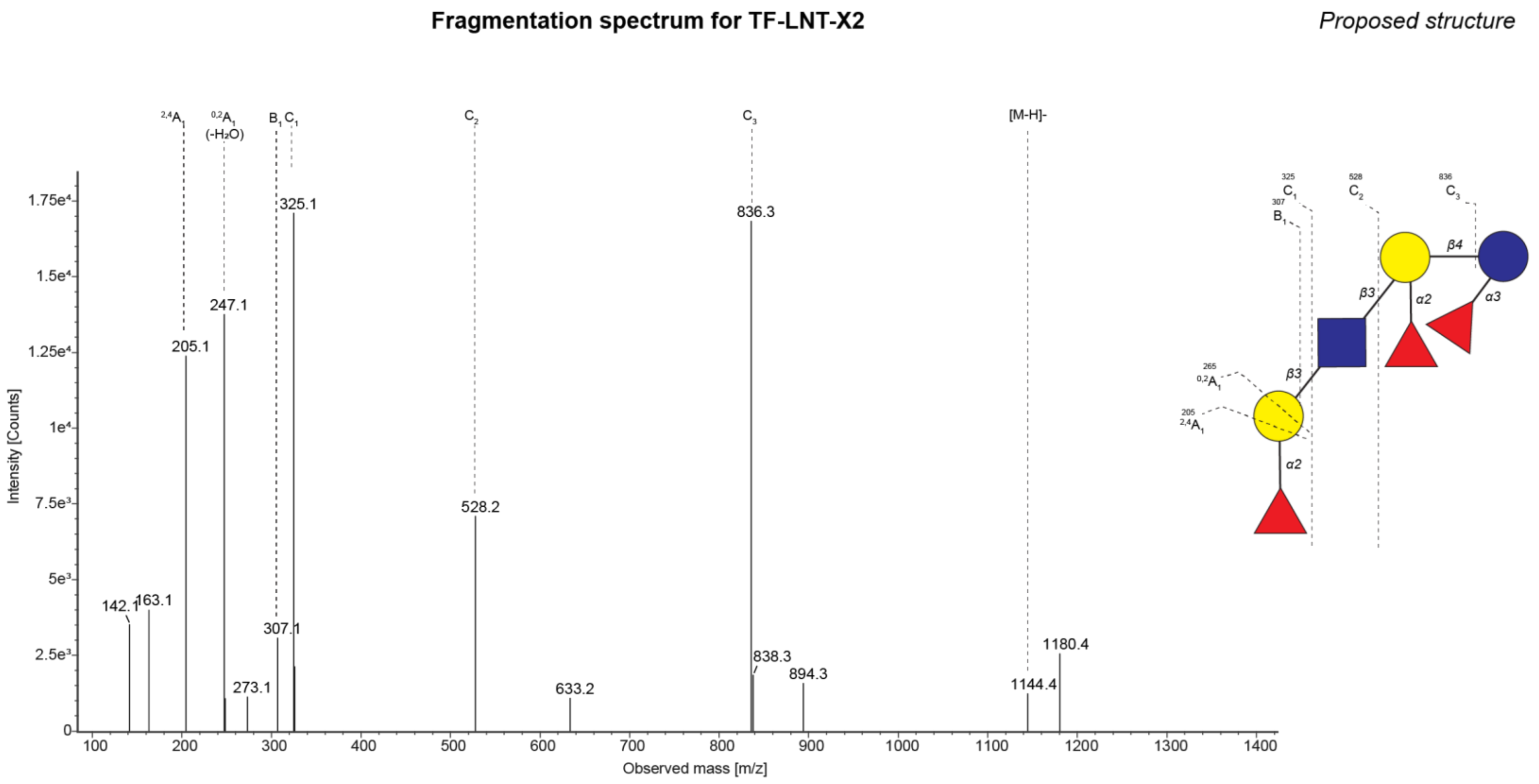

Figure S12. Retention- and drift time deconvoluted fragmentation spectrum for TF-LNT-X2. Spectrum was obtained analyzing the calibration reference sample upon long method LC-ESI-IMS-MS analysis. CID fragmentation energy was ramped from 30 to 70 eV for all ions fragmentation (AIF). In the spectrum, A, B, and C fragments are annotated over the peaks following Domon & Costello's nomenclature[6], while D-fragments, resulting from a consecutive fragmentation into a C fragment and Z fragment, are annotated as proposed by Chai, Piskarev & Lawson[7,8]. Fragment ions not measured in the spectrum on D-type fragmentation are annotated in gray. When relevant, water losses (-H_2O) are annotated next to their corresponding fragment ion by a dotted line. The structure of this isomer was originally unknown (isomer X2 of the TF-LNTseries) but based on the spectral information a structure can be predicted and it is annotated to the right of the spectrum, together with the fragment ions observed at each monosaccharide residue. Monosaccharide symbols and structural representations of HMOs were drawn in Illustrator according to the Consortium for Functional Glycomics[9]. Linkage type (α or β) together with linkage position is annotated directly on the structure.

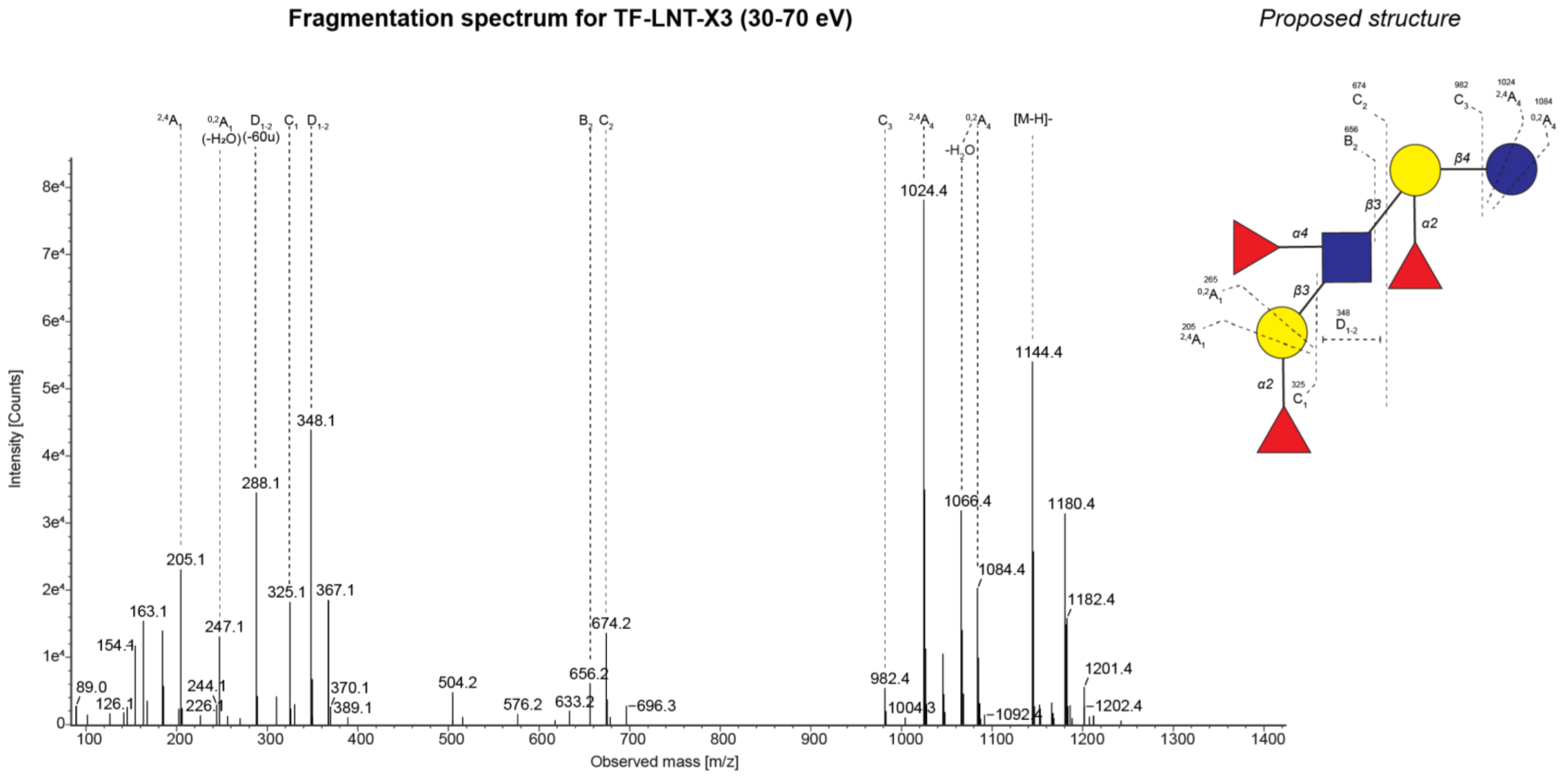

Figure S13. Retention- and drift time deconvoluted fragmentation spectrum for TF-LNT-X3. Spectrum was obtained analyzing the calibration reference sample using the long method LC-ESI-IMS-MS workflow. CID fragmentation energy was ramped from 30 to 70 eV for all ions fragmentation (AIF). In the spectrum, A, B, and C fragments are annotated over the peaks following Domon & Costello's nomenclature[6] while D-fragments, resulting from a consecutive fragmentation into a C fragment and Z fragment, are annotated as proposed by Chai, Piskarev & Lawson[7,8]. Fragment ions not measured in the spectrum on D-type fragmentation are annotated in gray. When relevant, water losses (-H_2O) are annotated next to their corresponding fragment ion by a dotted line. The structure of this isomer was originally unknown (isomer X3 of the TF-LNT series) but based on the spectral information a structure can be predicted and it is annotated to the right of the spectrum, together with the fragment ions observed at each monosaccharide residue. Monosaccharide symbols and structural representations of HMOs were drawn in Illustrator according to the Consortium for Functional Glycomics[9]. Linkage type (α or β) together with linkage position is annotated directly on the structure.

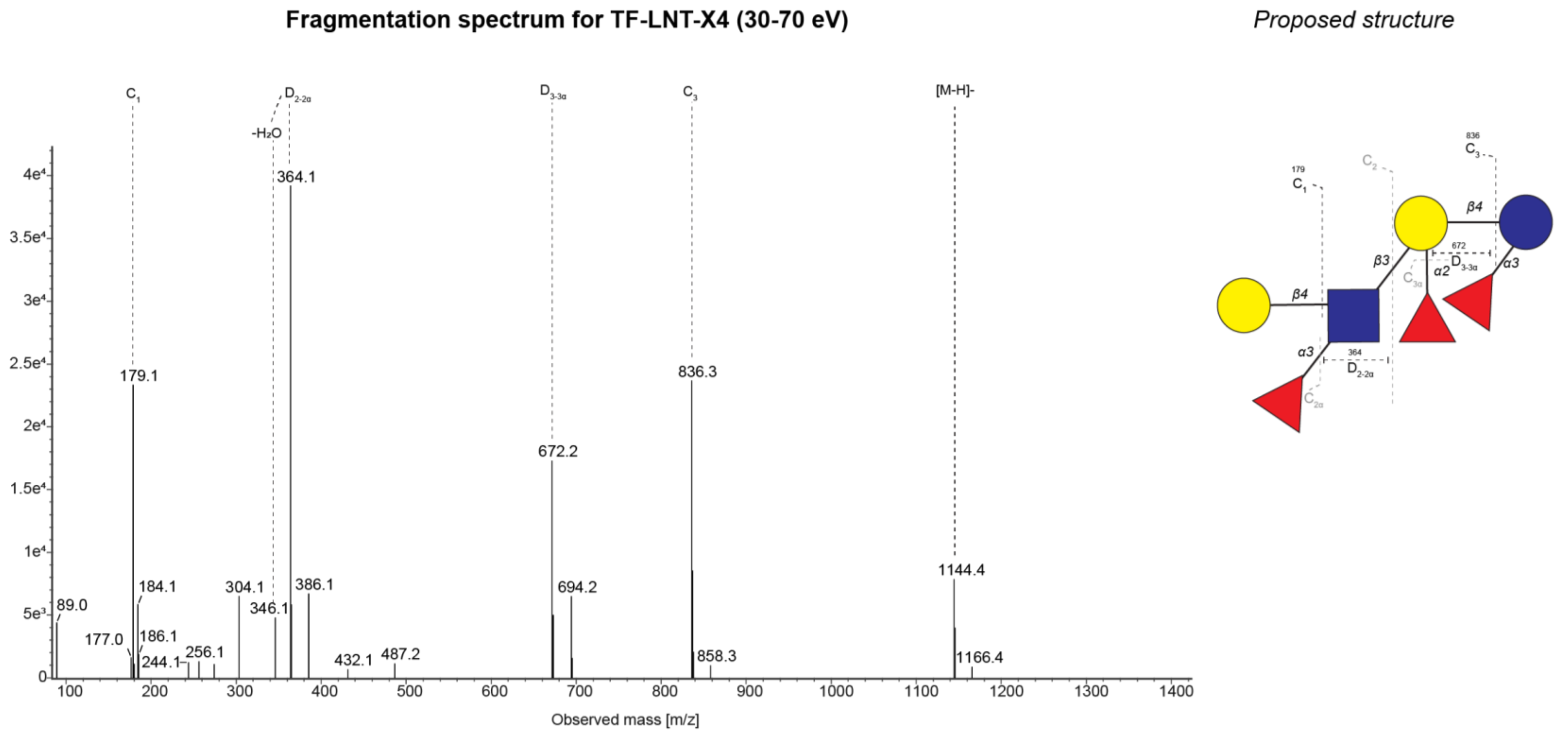

Figure S14. Retention- and drift time deconvoluted fragmentation spectrum for TF-LNT-X4. Spectrum was obtained analyzing the calibration reference sample using the long method LC-ESI-IMS-MS workflow. CID fragmentation energy was ramped from 30 to 70 eV for all ions fragmentation (AIF). In the spectrum, A, B, and C fragments are annotated over the peaks following Domon & Costello's nomenclature[6] while D-fragments, resulting from a consecutive fragmentation into a C fragment and Z fragment, are annotated as proposed by Chai, Piskarev & Lawson[7,8]. Fragment ions not measured in the spectrum on D-type fragmentation are annotated in gray. When relevant, water losses (-H_2O) are annotated next to their corresponding fragment ion by a dotted line. The structure of this isomer was originally unknown (isomer X4 of the TF-LNT series) but based on the spectral information a structure can be predicted and it is annotated to the right of the spectrum, together with the fragment ions observed at each monosaccharide residue. Monosaccharide symbols and structural representations of HMOs were drawn in Illustrator according to the Consortium for Functional Glycomics[9]. Linkage type (α or β) together with linkage position is annotated directly on the structure.

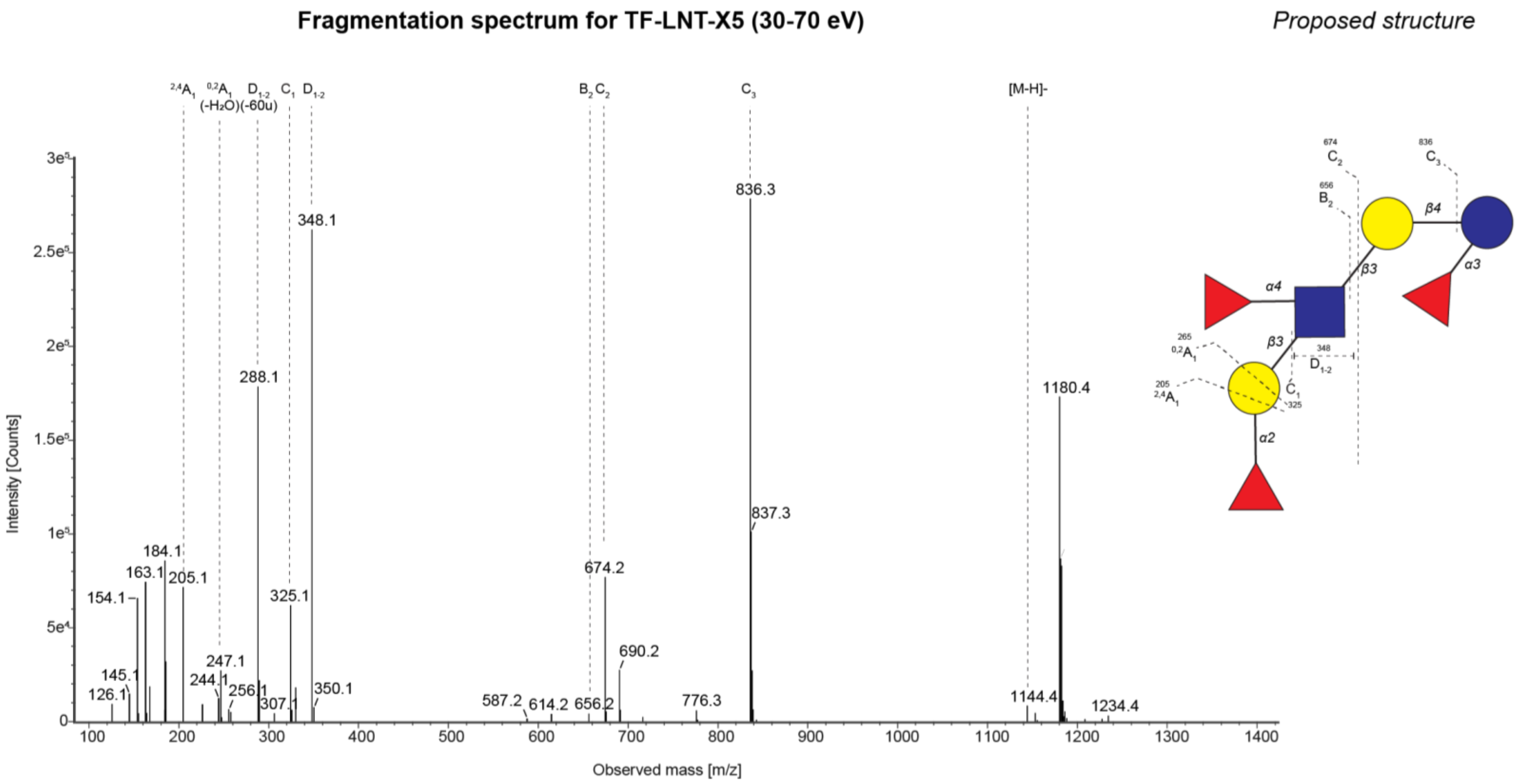

Figure S15. Retention- and drift time deconvoluted fragmentation spectrum for TF-LNT-X5. Spectrum was obtained analyzing the calibration reference sample using the long method LC-ESI-IMS-MS workflow. CID fragmentation energy was ramped from 30 to 70 eV for all ions fragmentation (AIF). In the spectrum, A, B, and C fragments are annotated over the peaks following Domon & Costello's nomenclature[6] while D-fragments, resulting from a consecutive fragmentation into a C fragment and Z fragment, are annotated as proposed by Chai, Piskarev & Lawson[7,8]. Fragment ions not measured in the spectrum on D-type fragmentation are annotated in gray. When relevant, water losses (-H_2O) are annotated next to their corresponding fragment ion by a dotted line. The structure of this isomer was originally unknown (isomer X5 of the TF-LNT series) but based on the spectral information a structure can be predicted and it is annotated to the right of the spectrum, together with the fragment ions observed at each monosaccharide residue. Monosaccharide symbols and structural representations of HMOs were drawn in Illustrator according to the Consortium for Functional Glycomics[9]. Linkage type (α or β) together with linkage position is annotated directly on the structure.

5. Fragmentation spectra of LST a

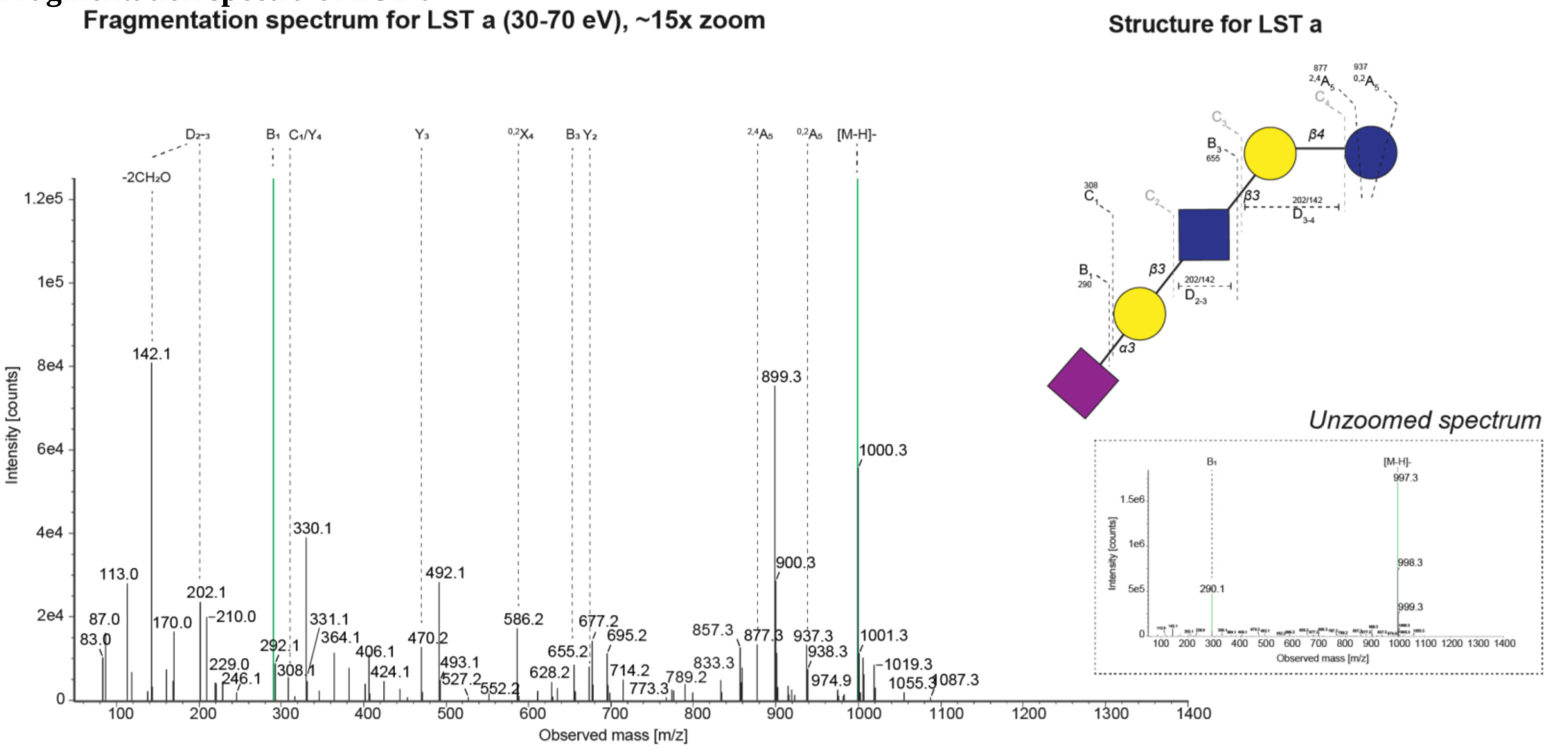

Figure S16. Retention- and drift time deconvoluted fragmentation spectrum for LST a. Spectrum was obtained analyzing the calibration reference sample using the long method LC-ESI-IMS-MS workflow. CID fragmentation energy was ramped from 30 to 70 eV for all ions fragmentation (AIF). The spectrum displayed is zoomed about 15x, and an unzoomed insert is presented on the right of the spectrum, peaks highlighted in green correspond in both spectra. In the zoomed spectrum, A, B, and C fragments are annotated over the peaks following Domon & Costello's nomenclature[6] while D-fragments, resulting from a consecutive fragmentation into a C fragment and Z fragment, are annotated as proposed by Chai, Piskarev & Lawson[7,8,12.] Fragment ions not measured in the spectrum on D-type fragmentation are annotated in gray. Monosaccharide symbols and structural representations of HMOs were drawn in Illustrator according to the Consortium for Functional Glycomics[9]. Linkage type (α or β) together with linkage position is annotated directly on the structure.

6. S-curves of HMO abundance over the course of lactation

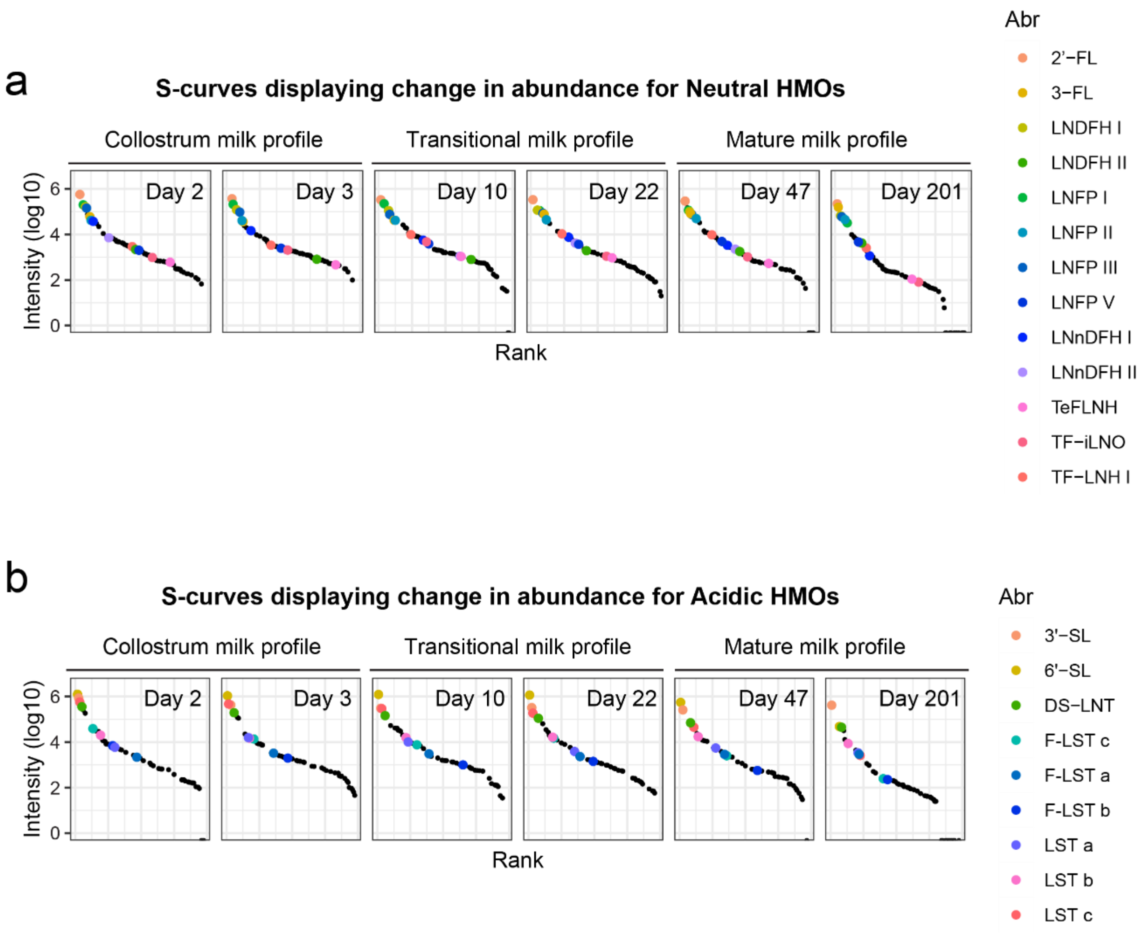

Figure S17. S-curves displaying the changes in the HMO profiles over lactation as measured in milk from a Type-I donor analyzed using the short method LC-ESI-IMS-MS workflow. S-curves represent ranked intensity of different HMOs. Acidic and Neutral HMOs are separated to prevent biases due to better ionization observed for acidic HMOs in the negative mode ESI. Some selected HMOs are highlighted to illustrate their variability over time, color gradient per selected HMO is depicted to the right of the s-curves.

7. Supplementary Tables

Table S1. **The commercial HMO standards used are listed**. Compound name, Abbreviation, Degree of polymerization (DP), Retention time (RT) elution in the short and long method HILIC-Amide LC-ESI-IMS-MS workflows, Supplier, Catalogue number and Structure (IUPAC condensed formula) are provided. Compounds are classified based on their charge (Neutral HMOs or Acidic HMOs).

Component name	Abbreviation	Degree of polymerization	Retention time (short method)	Retention time (long method)	Supplier	Catalogue number	Structure
Neutral HMOs							
2'-Fucosyllactose	2'-FL	3	14.3	16.55	IsoSep	35/08	Fuc(a1-2)Gal(b1-4)Glc
3-Fucosyllactose	3-FL	3	15.4	18.15	IsoSep	35/09	Fuc(a1-3)[Gal(b1-4)]Glc
3'-Galactosyllactose	ß3'-GL	3	18.5	23.15	Carbosynth	OG15069	Gal(b1-3)Gal(b1-4)Glc
6'-Galactosyllactose	ß6'-GL	3	18.2	22.7	Carbosynth	OG15070	Gal(b1-6)Gal(b1-4)Glc
a-Tetrasaccharide	a-Tetra	4	18.8	23.85	IsoSep	45/05	Fuc(a1-2)[GalNac(a1-3)]Gal(b1-4)Gal
Difucosyllactose	DFL	4	18.8	23.6	Isosep	45/02	Fuc(a1-2)Gal(b1-4)[Fuc(a1-3)]Glc
Lacto-N-tetraose	LNT	4	22	29.65	IsoSep	45/01	Gal(b1-3)GlcNAc(b1-3)Gal(b1-4)Glc
Lacto-N-neo-tetraose	LNnT	4	22.4	30.45	IsoSep	45/08	Gal(b1-4)GlcNAc(b1-3)Gal(b1-4)Glc
a-Pentasaccharide	a-Penta	5	24.15	33.5	IsoSep	55/01	GalNAc(a1-3)[Fuc(a1-2)]Gal(b1-4)[Fuc(a1-3)]Glc
b-Pentasaccharide	b-Penta	5	27.15	39.15	IsoSep	55/02	Gal(a1-3)[Fuc(a1-2)]Gal(b1-4)[Fuc(a1-3)]Glc
Lacto-N-neo-fucopentaose I	LNnFP-I	5	24.95	35.3	Carbosynth	OL09903	Fuc(a1-2)Gal(b1-4)GlcNAc(b1-3)Gal(b1-4)Glc
Lacto-N-fucopentaose I	LNFP-I	5	25.9	37.1	IsoSep	55/05	Fuc(a1-2)Gal(b1-3)GlcNAc(b1-3)Gal(b1-4)Glc
Lacto-N-fucopentaose II	LNFP-II	5	27.65	40.5	IsoSep	55/06	Gal(b1-3)[Fuc(a1-4)]GlcNAc(b1-3)Gal(b1-4)Glc
Lacto-N-fucopentaose III	LNFP-III	5	27.4	40	IsoSep	55/07	Gal(b1-4)[Fuc(a1-3)]GlcNAc(b1-3)Gal(b1-4)Glc
Lacto-N-fucopentaose V	LNFP-V	5	26.6	38.45	IsoSep	55/08	Gal(b1-3)GlcNAc(b1-3)Gal(b1-4)[Fuc(a1-3)]Glc
Lacto-N-fucopentaose VI	LNFP-VI	5	27.05	39.3	Dextra	L514	Gal(b1-4)GlcNAc(b1-3)Gal(b1-4)[Fuc(a1-3)]Glc
Lacto-N-neo-difucohexaose I	LNnDFH-I	6	30.35	46.05	ChemilyBio	HMO-1016	Fuc(a1-2)Gal(b1-4)[Fuc(a1-3)]GlcNAc(b1-3)Gal(b1-4)Glc
Lacto-N-neo-difucohexaose II	LNnDFH-II	6	31.75	49.1	ChemilyBio	GO-2022	Gal(b1-4)[Fuc(a1-3)]GlcNAc(b1-3)Gal(b1-4)[Fuc(a1-3)]Glc
Lacto-N-difucohexaose I	LNDFH-I	6	32.05	49.55	IsoSep	56/03	Fuc(a1-2)Gal(b1-3)[Fuc(a1-4)]GlcNAc(b1-3)Gal(b1-4)Glc
Lacto-N-difucohexaose II	LNDFH-II	6	32.2	49.8	Carbosynth	OL06826	Gal(b1-3)[Fuc(a1-4)]GlcNAc(b1-3)Gal(b1-4)[Fuc(a1-3)]Glc
Lacto-N-hexaose	LNH	6	31.95	50	Carbosynth	OL02436	Gal(b1-3)GlcNAc(b1-3)[Gal(b1-4)GlcNAc(b1-6)]Gal(b1-4)Glc
Lacto-N-neo-hexaose	LNnH	6	32.25	50.5	Agilent Technologies	GKAD-01024	Gal(b1-4)GlcNAc(b1-3)[Gal(b1-4)GlcNAc(b1-6)]Gal(b1-4)Glc
Lacto-para-N-neo-hexaose	pLNnH	6	31.9	50.15	Carbosynth	OL46818	Gal(b1-4)GlcNAc(b1-3)Gal(b1-4)GlcNAc(b1-3)Gal(b1-4)Glc
a-Heptasaccharide	a-Hepta	7	35.05	56.55	IsoSep	57/01	GalNAc(a1-3)[Fuc(a1-2)]Gal(b1-3)[Fuc(a1-4)]GlcNAc(b1-3)Gal(b1-4)Glc
Fucosyllacto-para-N-hexaose I	F-pLNH-I	7	36.25	60.35	Glycoseparations	LE7-4	Gal(b1-3)GlcNAc(b1-3)Gal(b1-4)[Fuc(a1-3)]GlcNAc(b1-3)Gal(b1-4)Glc
Fucosyllacto-N-hexaose II	F-LNH-II	7	35.55	59.35	Glycoseparations	LE7-8	Gal(b1-3)GlcNAc(b1-3)[Gal(b1-4)[Fuc(a1-3)]GlcNAc(b1-6)]Gal(b1-4)Glc
Difucosyl-lacto-N-neo-hexaose I	DF-LNnH-I	8	39.6	67.9	IsoSep	57/13	Gal(b1-4)[Fuc(a1-3)]GlcNAc(b1-3)[Gal(b1-4)[Fuc(a1-3)]GlcNAc(b1-6)]Gal(b1-4)Glc
Difucosyl-lacto-para-N-hexaose I	DF-pLNH-I	8	40.65	68.65	IsoSep	57/15	Gal(b1-3)[Fuc(a1-4)]GlcNAc(b1-3)Gal(b1-4)[Fuc(a1-3)]GlcNAc(b1-3)Gal(b1-4)Glc
Difucosyl-lacto-N-hexaose I	DF-LNH-I	8	38.5	64.2	IsoSep	57/14	Fuc(a1-2)Gal(b1-3)GlcNAc(b1-3)[Gal(b1-4)[Fuc(a1-3)]GlcNAc(b1-6)]Gal(b1-4)Glc
Difucosyl-lacto-N-hexaose II	DF-LNH-II	8	39.85	66.55	IsoSep	57/17	Gal(b1-3)[Fuc(a1-4)]GlcNAc(b1-3)[Gal(b1-4)[Fuc(a1-3)]GlcNAc(b1-6)]Gal(b1-4)Glc
Lacto-N-neo-octaose	LNnO	8	39.45	66.9	Carbosynth	OL09902	Gal(b1-4)GlcNAc(b1-3)Gal(b1-4)GlcNAc(b1-3)Gal(b1-4)GlcNAc(b1-3)Gal(b1-4)Glc
Fucosyl(1-3)-iso-lacto-N-octaose	F-iLNO	9	42.4	73.55	Glycoseparations	LE9-4	Gal(b1-3)GlcNAc(b1-3)Gal(b1-4)[Fuc(a1-3)]GlcNAc(b1-6)[Gal(b1-3)GlcNAc(b1-3)]Gal(b1-4)Glc
Trifucosyllacto-N-hexaose I	TF-LNH-I	9	43.2	74.7	Isosep	57/18	Fuc(a1-2)Gal(b1-3)[Fuc(a1-4)]GlcNAc(b1-3)[Gal(b1-4)[Fuc(a1-3)]GlcNAc(b1-6)]Gal(b1-4)Glc
Trifucosyl(1-2,1-2,1-3)-iso-lacto-N-octaose	TF-iLNO	11	47.1	84.4	Biosynth Carbosynth	OT13725	Fuc(a1-2)Gal(b1-3)GlcNAc(b1-3)Gal(b1-4)[Fuc(a1-3)]GlcNAc(b1-6)[Fuc(a1-2)Gal(b1-3)GlcNAc(b1-3)]Gal(b1-4)Glc
Acidic HMOs							
Lactose-3'-Sulfate	L-3'S	2	11.5	12.9	TRC	L165225	Gal3S(b1-4)Glc
3'-sialyllactose	3'-SL	3	20.45	27.1	IsoSep	35/01	Neu5Ac(a2-3)Gal(b1-4)Glc
6'-sialyllactose	6'-SL	3	22.9	31.65	Carbosynth	OS04398	Neu5Ac(a2-6)Gal(b1-4)Glc
6'-Sialyl-N-acetyllactosamine	6'-SLN	3	19.7	25.75	Sigma-Aldrich	37966	Neu5Ac(b2-6)Gal(b1-4)GlcNAc
3'-Sialyl-3-fucosyllactose	3'-S-3-FL	4	24.8	35.35	Isosep	45/50-0005	Neu5Ac(a2-3)Gal(b1-4)[Fuc(a1-3)]Glc
Sialyllacto-N-tetraose a	LSTa	5	30.2	46.85	IsoSep	55/50	Neu5Ac(a2-3)Gal(b1-3)GlcNAc(b1-3)Gal(b1-4)Glc
Sialyllacto-N-tetraose b	LSTb	5	31.45	49.25	IsoSep	55/51	Neu5Ac(a2-6)Gal(b1-3)GlcNAc(b1-3)Gal(b1-4)Glc
Sialyllacto-N-tetraose c	LSTc	5	32.6	51.75	IsoSep	55/52	Neu5Ac(a2-6)Gal(b1-4)GlcNAc(b1-3)Gal(b1-4)Glc
Disialyllacto-N-tetraose	DS-LNT	6	38.15	64.8	Isosep	56/50	Neu5Ac(a2-3)Gal(b1-3)[Neu5Ac(a2-6)]GlcNAc(b1-3)Gal(b1-4)Glc
Fucosylsialyllacto-N-neo-tetraose c	F-LSTc	6	36.4	60.15	ChemilyBio	HMO-1023	Neu5Ac(a2-6)Gal(b1-4)GlcNAc(b1-3)Gal(b1-4)[Fuc(a1-3)]Glc
Fucosylsialyllacto-N-tetraose a	F-LSTa	6	35.6	58.3	IsoSep	56/51	Neu5Ac(a2-3)Gal(b1-3)[Fuc(a1-4)]GlcNAc(b1-3)Gal(b1-4)Glc
Fucosylsialyllacto-N-tetraose b	F-LSTb	6	34.7	56.35	IsoSep	56/52	Fuc(a1-2)Gal(b1-3)[Neu5Ac(a2-6)]GlcNAc(b1-3)Gal(b1-4)Glc

Table S2. **The measured HMOs per method are listed.** Compound name, Annotation code (as described in Remoroza et al.[10]), Degree of polymerization, Charge (where N=Neutral and A=Acidic), and for each method (including short and long runs on HILIC-Amide and long run on PGC) the following parameters are listed: isomer count, retention time (at mid-point of the isomer elution RT range) and elution range (in +/- min from mid-point).

				HILIC-Amide Column						PGC column		
				short method			long method			long method		
			Total Isomers	133			203			197		
Compound name	Annotation code #	Degree of polymerization *	Charge	Isomer count**	Retention time (min. at mid point)	Elution range (± min.)	Isomer count**	Retention time (min. at mid point)	Elution range (± min.)	Isomer count**	Retention time (min. at mid point)	Elution range (± min.)
Lactose-Sulfate	A2000S	2	A	2	13.0	2.0	2	12.5	1.5	2	11.0	2.0
Fucosyllactose	N2100	3	N	7	14.0	3.0	7	17.5	1.5	7	23.5	16.5
Sialyllactose	A2001	3	A	2	23.0	2.0	2	29.5	3.5	2	20.0	5.0
Fucosyllactose-sulfate	A2100S	3	A	1	17.0	0.0	1	21.2	0.0	2	25.0	1.0
Fucosylsialyllactose	A2101	4	A	1	25.5	0.5	1	35.0	1.0	1	20.0	1.0
Difucosyllactose	N2200	4	N	5	17.5	2.5	5	20.5	3.5	1	19.5	0.5
Lacto-N-tetratose	N3010	4	N	2	23.0	1.0	2	29.5	1.5	2	43.5	1.5
Sialyllacto-N-tetraose	A3011	5	A	3	32.5	1.5	3	49.5	3.5	3	44.5	2.5
Fucosyllacto-N-tetraose (LNFP)	N3110	5	N	6	27.5	1.5	7	38.0	5.0	6	37.5	7.5
Disialyllacto-N-tetraose	A3012	6	A	3	39.0	2.0	3	66.0	5.0	1	47.0	1.0
Fucosylsialyllacto-N-tetraose	A3111	6	A	5	36.0	2.0	5	57.0	4.0	5	38.5	6.5
Difucosyllacto-N-tetraose (LNDFH)	N3210	6	N	6	31.0	2.0	6	45.5	4.5	4	25.5	5.5
Lacto-N-hexaose	N4020	6	N	4	33.0	1.0	4	49.5	1.5	5	68.5	6.5
Difucosylsialyllacto-N-tetraose	A3211	7	A	3	40.0	1.0	4	66.5	1.5	*n.d.*	*n.d.*	*n.d.*
Fucosyldisialyllacto-N-tetraose	A3112	7	A	3	42.0	2.0	3	71.5	4.5	*n.d.*	*n.d.*	*n.d.*
Sialyllacto-N-hexaose	A4021	7	A	2	41.0	1.0	2	68.5	1.5	2	60.0	2.0
Fucosyllacto-N-hexaose	N4120	7	N	5	36.5	1.5	8	58.0	3.0	*11*	*59.5*	*10.5*
Trifucosyllacto-N-tetraose	N3310	7	N	5	35.5	2.5	5	55.5	4.5	5	22.5	7.5
Disialyllacto-N-Hexaose	A4022	8	A	5	46.0	2.0	6	80.0	4.0	1	58.0	1.0
Fucosylsialyllacto-N-Hexaose	A4121	8	A	6	43.0	2.0	6	73.5	4.5	6	51.0	6.0
Difucosyllacto-N-hexaose	N4220	8	N	7	40.5	1.5	9	65.0	5.0	10	46.0	11.0
Lacto-N-octaose	N5030	8	N	3	40.0	1.0	3	66.5	1.5	3	85.5	4.5
Difucosylsialyllacto-N-hexaose	A4221	9	A	4	46.5	1.5	4	80.5	2.5	1	46.0	1.0
Fucosyldisialyllacto-N-hexaose	A4122	9	A	2	47.0	1.0	2	83.0	2.0	3	47.5	7.5
Sialyllacto-N-octaose	A5031	9	A	1	46.5	0.5	1	82.0	1.0	*n.d.*	*n.d.*	*n.d.*
Fucosyllacto-N-octaose	N5130	9	N	6	43.5	1.5	8	74.0	3.0	11	72.0	13.0
Trifucosyllacto-N-hexaose	N4320	9	N	5	43.0	2.0	5	73.0	5.0	17	41.5	15.5
Trisialyllacto-N-hexaose	N4023	9	N	1	49.5	0.5	1	89.0	1.0	*n.d.*	*n.d.*	*n.d.*
Difucosyldisialyllacto-N-hexaose	A4222	10	A	3	49.0	2.0	3	87.5	3.5	*n.d.*	*n.d.*	*n.d.*
Fucosylsialyllacto-N-octaose	A5131	10	A	6	49.0	2.0	7	86.0	4.0	3	65.0	7.0
Difucosyllacto-N-octaose	N5230	10	N	4	46.0	2.0	11	80.0	4.0	24	61.5	15.5
Lacto-N-decaose	N6040	10	N	3	46.5	1.5	6	82.0	3.0	1	95.0	1.0
Tetrafucosyllacto-N-hexaose	N4420	10	N	6	46.0	2.0	6	80.0	5.0	7	35.5	9.5
Fucosyllacto-N-decaose	N6140	11	N	*n.d.*	*n.d.*	*n.d.*	7	86.0	3.0	7	82.5	7.5
Trifucosyllacto-N-octaose	N5330	11	N	6	49.0	2.0	13	87.0	4.0	18	52.5	12.5
Difucosyldisialyllacto-N-octaose	A5232	12	A	*n.d.*	*n.d.*	*n.d.*	4	98.0	2.0	*n.d.*	*n.d.*	*n.d.*
Difucosyllacto-N-Decaose	N6240	12	N	*n.d.*	*n.d.*	*n.d.*	11	91.0	5.0	13	72.5	12.5
Tetrafucosyllacto-N-Octaose	N5430	12	N	*n.d.*	*n.d.*	*n.d.*	6	94.0	4.0	7	50.5	7.5
Fucosyllacto-N-dodecaose	N7150	13	N	*n.d.*	*n.d.*	*n.d.*	8	96.5	3.5	*n.d.*	*n.d.*	*n.d.*
Trifucosyllacto-N-decaose	N6340	13	N	*n.d.*	*n.d.*	*n.d.*	6	96.0	4.0	6	62.5	14.5

(*) Hexose, Fucose, GlcNac and Neu5Ac are counted as DP

(**) Isomers were counted visualy in the EIC of the compounds of the 2.5 mg/ml injection of isolated fractions

(#) Annotation code - A2000S denotes A=Acidic or N=Neutral, 2 Hexoses, 0 Fucoses, 0 GlcNAc, 0 Neu5Ac and S=Sulfate.

8. References

(1) Auer, F., Jarvas, G. & Guttman, A. Recent advances in the analysis of human milk oligosaccharides by liquid phase separation methods. *J Chromatogr B Analyt Technol Biomed Life Sci* **1162**, 122497 (2021).

(2) Cao, C. *et al.* Isolation of Human Milk Difucosyl Nona- and Decasaccharides by Ultrahigh-Temperature Preparative PGC-HPLC and Identification of Novel Difucosylated Heptaose and Octaose Backbones by Negative-Ion ESI-MSn. *Anal Chem* **96**, 6170–6179 (2024).

(3) Fountain, K. J., Hudalla, C. J., Mccabe, D. R. & Morrison, D. Analysis of Carbohydrates by UltraPerformance Liquid Chromatography and Mass Spectrometry. *Application Note, Waters Corporation* https://www.waters.com/nextgen/no/en/library/application-notes/2009/analysis-of-carbohydrates-by-uplc-and-mass-spectrometry.html (2009).

(4) Hou, H. *et al.* Evaluation of Prebiotic Glycan Composition in Human Milk and Infant Formula: Profile of Galacto-Oligosaccharides and Absolute Quantification of Major Milk Oligosaccharides by UPLC-Cyclic IM-MS and UPLC-MS/MS. *J Agric Food Chem* **72**, 7980–7990 (2024).

(5) Russo, M. *et al.* Principles and applications of porous graphitic carbon stationary phase in liquid chromatography: An update. *J Chromatogr A* **1719**, 464728 (2024).

(6) Domon, B. & Costello, C. E. A systematic nomenclature for carbohydrate fragmentations in FAB-MS/MS spectra of glycoconjugates. *Glycoconj J* **5**, 397–409 (1988).

(7) Chai, W., Lawson, A. M. & Piskarev, V. Branching pattern and sequence analysis of underivatized oligosaccharides by combined MS/MS of singly and doubly charged molecular ions in negative-ion electrospray mass spectrometry. *J Am Soc Mass Spectrom* **13**, 670–679 (2002).

(8) Chai, W., Piskarev, V. & Lawson, A. M. Negative-Ion Electrospray Mass Spectrometry of Neutral Underivatized Oligosaccharides. *Anal Chem* **73**, 651–657 (2001).

(9) Varki, A. *et al.* Symbol nomenclature for glycan representation. *Proteomics* **9**, 5398 (2009).

(10) Remoroza, C. A., Mak, T. D., De Leoz, M. L. A., Mirokhin, Y. A. & Stein, S. E. Creating a Mass Spectral Reference Library for Oligosaccharides in Human Milk. *Anal Chem* **90**, 8977–8988 (2018).

(11) Urashima, T., Hirabayashi, J., Sato, S. & Kobata, A. Human milk oligosaccharides as essential tools for basic and application studies on galectins. *Trends in Glycoscience and Glycotechnology* **30**, SE51–SE65 (2018).

(12) Chai, W. *et al.* Analysis of chain and blood group type and branching pattern of sialylated oligosaccharides by negative ion electrospray tandem mass spectrometry. *Anal Chem* **78**, 1581–1592 (2006).
